# Machine learning for predicting distant metastasis in nasopharyngeal carcinoma patients

**DOI:** 10.3389/fimmu.2025.1580200

**Published:** 2025-06-05

**Authors:** Hong Sun, Jijie Zhu, Ling Li, Xiu Xin, Jingchao Yan, Taomin Huang

**Affiliations:** ^1^ Department of Pharmacy, Eye & ENT Hospital, Fudan University, Shanghai, China; ^2^ Shanghai University of Medicine & Health Sciences, Shanghai, China

**Keywords:** nasopharyngeal carcinoma, machine learning, distant metastasis, predictive model, immunotherapy, targeted therapy

## Abstract

**Background:**

Distant metastasis is the main cause of treatment failure and death in patients with nasopharyngeal carcinoma (NPC). The aim of this study was to explore the risk factors for distant metastasis in NPC patients using machine learning (ML) methods.

**Methods:**

We collected data from NPC patients who were treated at the Eye Ear Nose Throat Hospital of Fudan University between September 2017 and June 2024. Seven ML methods were employed to construct the predictive models. By comparing the predictive performance of different ML models, the best one was selected to establish a predictive model for distant metastasis of NPC. The SHapley Additive exPlanation (SHAP) method was utilized to ascertain the ranking of feature importance and to provide explanations for the predictive model.

**Results:**

A total of 1,845 NPC patients were included in this study. Among the seven models, Logistic Regression (LR) performed best in the test dataset (Area Under the ROC Curve [AUC] = 0.8499). SHAP analysis indicated that the most important variables for distant metastasis in NPC patients were targeted therapy, immunotherapy, N stage, Epstein-Barr virus (EBV), hypertension, T stage, lymphocyte count (LY) and lactate dehydrogenase (LDH) level.

**Conclusion:**

Targeted therapy, N stage, immunotherapy, EBV, hypertension, T stage, LY and LDH level are significantly associated with the risk of distant metastasis in NPC and could be used to identify high-risk populations for distant metastasis in NPC patients. For high-risk patients, early interventions such as targeted therapy and immunotherapy might be considered to reduce the risk of distant metastasis in NPC.

## 1 Introduction

Nasopharyngeal carcinoma (NPC), a subset of head and neck cancers, originates from the epithelial cells of the nasopharynx (1, 2). The development of NPC is associated with a variety of factors, including genetic susceptibility, infection by the Epstein-Barr virus (EBV), and environmental factors such as smoking (3–8). NPC has significant geographical differences, being prevalent in East and Southeast Asia (9). The early treatment of NPC mainly relies on radiotherapy and chemotherapy, which has a good prognosis (10). However, most patients were diagnosed at advanced stages with a poor prognosis. Patients with advanced NPC have a higher risk of distant metastasis. Over the past few decades, the survival rate of patients with locally advanced NPC has improved through successful chemoradiation strategies. Despite advances in treatment strategies, approximately 30% of NPC patients still experience recurrence or metastatic disease (11). Distant metastasis is the main cause of treatment failure and death in NPC patients (12).

Patients with metastatic NPC are usually advised to undergo platinum-based chemotherapy as first-line treatment. However, while the combination of gemcitabine and cisplatin has been frequently utilized in recent years, it has been found to offer only limited short-term benefits. Although Programmed Cell Death Protein 1 (PD-1) and Programmed Cell Death Ligand 1 (PD-L1) inhibitors have brought new hope to metastatic NPC patients in recent years, there are still some challenges and limitations, such as drug resistance and economic burden associated with long-term use (13, 14). Moreover, although tislelizumab plus chemotherapy appeared to be the optimal choice compared with other PD-1 inhibitors plus chemotherapy for the first-line treatment of recurrent or metastatic nasopharyngeal carcinoma, there were still limited benefit in many patients (15, 16). Overall, the prognosis for patients with metastatic NPC is poor. Therefore, exploring the risk factors affecting distant metastasis in NPC and constructing a prediction model for distant metastasis in NPC is important for improving the prognosis of NPC patients. In this study, we aimed to construct and validate a machine learning (ML) model to predict the risk of distant metastasis in NPC patients. The SHapley Additive exPlanation (SHAP) method was used to elucidate the feature importance and interpret the model’s predictive results, thereby assessing the model’s practical utility in predicting the distant metastasis in NPC patients.

## 2 Materials and methods

### 2.1 Study design

**Figure 1:**
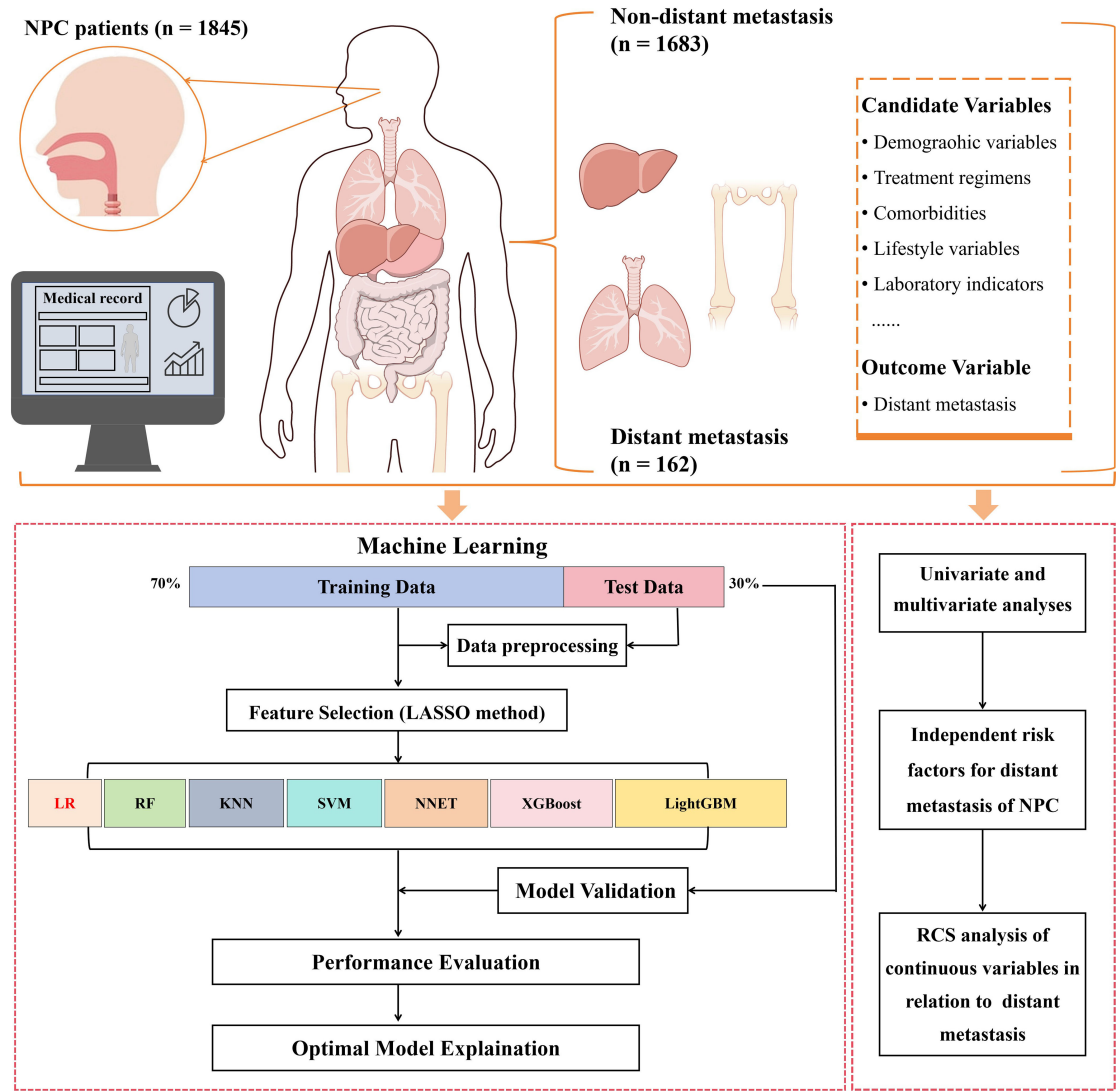
Study design of the study.

The study design was displayed in **Figure 1**. The data of 1,845 NPC patients was analyzed in this study. The candidate variables including demographic variables, treatment regimens, comorbidities, lifestyle variables, laboratory indicators and the outcome variable (distant metastasis) were collected. The dataset was split into training and test subsets with a ratio of 7:3. The Least Absolute Shrinkage and Selection Operator (LASSO) method was utilized on the training dataset to pinpoint the most significant features. Then, the most significant features were included in seven ML models. According to the performance of each ML model, the optimal model was selected to establish a predictive model for distant metastasis in NPC. The SHAP method was deployed to ascertain the ranking of feature importance and to elucidate the predictive model’s outcomes. The univariate and multivariate analyses were used on the entire cohort to identify the independent risk factors. The association between the independent continuous factors and distant metastasis was assessed through the application of a restricted cubic spline (RCS) model.

### 2.2 Study population

This study collected the data from patients at Eye Ear Nose Throat Hospital of Fudan University between September 2017 and June 2024. The criteria for participant inclusion were as follows: 1) patients were diagnosed as NPC by pathology; 2) metastatic lesions were diagnosed by imaging and pathology; 3) patients for whom demographic data, laboratory, imaging, pathological information and treatment regimens was recorded completely. Exclusion criteria were: 1) the data of laboratory, pathological, or imaging was absent; 2) patients with multiple primary malignancies. Ultimately, this study encompassed data from 1,845 NPC patients. Adhering to the regulations of China and principles of the Declaration of Helsinki, the research received approval from the Ethics Committee of the Eye Ear Nose Throat Hospital of Fudan University (2024222). This study was registered on ChiCTR (ChiCTR2500095104). Given the retrospective nature of the study and the anonymization of all data, the necessity for obtaining informed consent from the patients was waived.

### 2.3 Data collection

The data of variables analyzed in this research was sourced from the electronic medical records of patients in our hospital. The collected data were as follows: 1) demographic information: age, gender, and body mass index (BMI); 2) pathological information: Tumor Node Metastasis (TNM) stage (American Joint Committee on Cancer 8th edition), tumor differentiation, gene expression; 3) laboratory indicators (first set of tests after NPC admission): alanine aminotransferase (ALT), aspartate aminotransferase (AST), albumin (ALB), globulin (GLOB), blood urea nitrogen (BUN), creatinine (CREA), lactate dehydrogenase (LDH), white blood cell (WBC) count, hemoglobin (HGB) level, platelet (PLT) count, neutrophil count (NE), lymphocyte count (LY); 4) treatment regimens: radiotherapy, chemotherapy, targeted therapy, immunotherapy (If the patient had distant metastasis, only the treatment regimen before metastasis was collected); 5) comorbidities: hypertension, diabetes, hepatitis B, etc.; 6) lifestyle: smoking history, drinking history; 7) EBV infection.

### 2.4 Data processing and model building

Variables with missing data exceeding 10% were excluded. The proportion of missing data for each variable was displayed in the **Supplementary Figure S1**. The missing values were estimated by employing the random forest algorithm with its standard settings. Random forest imputation was chosen due to its ability to handle complex interactions between variables and its robustness in various missing data scenarios. In addition, we adopted multiple imputation for sensitivity analysis. The dataset was subsequently partitioned into a training set and a test set with a ratio of 7:3. LASSO method was performed to screen the significant features. Seven widely used ML methods were employed, including Logistic Regression (LR) (17), Random Forest (RF) (18), K-Nearest Neighbor (KNN) (19), Support Vector Machine (SVM) (20), Neural Network (NNET) (21), eXtreme Gradient Boosting (XGBoost) (22), and Light Gradient Boosting Machine (LightGBM) (23). Each of these ML models was trained by the training dataset and validated by the test set. We used the cross-validation to assess the performance of each model. Receiver operating characteristic curves (ROC) and decision curve analysis (DCA) were performed to assess the performance of different ML models. According to the performance of different ML models, the best model was selected to establish the predictive model. Subsequently, the SHAP method was implemented on the best-performing model to decipher the role of features and their clinical significance. The detailed code of data processing and model building was displayed in **Supplementary Materials**.

### 2.5 Statistical analysis

The ML models were established using R software (version 4.4.2) and related software packages such as “xgboost”, “lightgbm”, and “randomForest”. The discrimination performance of different ML models was evaluated by the analysis of the ROC curve. DCA was implemented to illustrate the net benefit of employing a model across various thresholds, thereby evaluating the clinical utility of the model. Continuous variables were expressed as the mean ± standard deviation (SD) and were analyzed using the t-test for comparisons. Categorical variables were displayed as numbers with their respective percentages and were assessed using the chi-square test for differences. The independent risk factors were identified through both univariate and multivariate logistic regression analyses. The association between the independent continuous factors and distant metastasis was evaluated using a RCS model. A two-tailed *P* value of less than 0.05 was considered statistically significant.

## 3 Results

### 3.1 Characteristics of study participants

A total of 1,845 NPC patients were analyzed in this study. Among them, 162 patients occurred distant metastasis. The cohort comprised 1,348 males (73.06%) and 497 females (26.94%). In terms of lifestyle factors, 812 individuals (44.01%) reported smoking, and 606 (32.85%) reported alcohol consumption. Concerning tumor stage and differentiation, the majority of patients presented with advanced T3/T4 stage (1,426, 77.29%), N2/N3 stage (1,176, 63.74%), and undifferentiated carcinoma (1,616, 87.59%). Regarding therapeutic approaches, 1,204 patients (65.26%) underwent targeted therapy, while 246 (13.33%) received immunotherapy. Almost all patients received radiotherapy or radiotherapy combined with chemotherapy. Comorbid conditions included hypertension in 507 patients (27.48%), diabetes in 156 (8.46%), hepatitis B in 61 (3.31%), and a history of other tumors in 44 (2.38%). Genetically, most patients exhibited positive expression for CKpan, P40, P63, EGFR, and EGER, whereas P16 expression was negative in the majority. In terms of EBV infection, 1,352 patients (73.28%) tested positive. The average age of the patients was 52.34 years old, and the average BMI was 23.81 kg/m².

### 3.2 Independent risk factors and dose-response relationship

We investigated the independent risk factors for distant metastasis in NPC patients. Through univariate logistic regression analysis, 11 potential risk factors were pinpointed to be significantly associated with distant metastasis in NPC (*P* < 0.05; **Table 1**). Subsequent multivariate logistic regression analysis revealed 9 factors that were independently associated with the risk of distant metastasis in NPC patients (*P* < 0.05; **Table 1**). These independent risk factors were gender, T stage, N stage, targeted therapy, immunotherapy, hypertension, EBV, LDH and LY.

**Table 1:** Results of the univariate and multivariate logistic regression analyses.

Characteristic	Non-distant metastasis (N=1683)	Distant metastasis (N=162)	OR (univariable)	OR (multivariable)
Gender, n (%)				
Female	465 (27.6%)	32 (19.8%)		
Male	1218 (72.4%)	130 (80.2%)	1.55 (1.04-2.32, *P*=0.032)	1.84 (1.18-2.86, *P*=0.007)
Smoking, n (%)				
No	950 (56.4%)	83 (51.2%)		
Yes	733 (43.6%)	79 (48.8%)	1.23 (0.89-1.70, *P*=0.202)	
Drinking, n (%)				
No	1132 (67.3%)	107 (66%)		
Yes	551 (32.7%)	55 (34%)	1.06 (0.75-1.48, *P*=0.754)	
T stage, n (%)				
T1/2	405 (24.1%)	14 (8.6%)		
T3/4	1278 (75.9%)	148 (91.4%)	3.35 (1.91-5.86, *P*<0.001)	3.14 (1.74-5.66, *P*<0.001)
N stage, n (%)				
	647 (38.4%)	22 (13.6%)		
N2/3	1036 (61.6%)	140 (86.4%)	3.97 (2.51-6.30, *P*<0.001)	3.38 (2.07-5.51, *P*<0.001)
Tumor differentiation, n (%)				
Undifferentiated	1474 (87.6%)	142 (87.7%)		
Differentiated	209 (12.4%)	20 (12.3%)	0.99 (0.61-1.62, *P*=0.979)	
Targeted therapy, n (%)				
No	543 (32.3%)	98 (60.5%)		
Yes	1140 (67.7%)	64 (39.5%)	0.31 (0.22-0.43, *P*<0.001)	0.28 (0.19-0.40, *P*<0.001)
Immunotherapy, n (%)				
No	1439 (85.5%)	160 (98.8%)		
Yes	244 (14.5%)	2 (1.2%)	0.07 (0.02-0.30, *P*<0.001)	0.05 (0.01-0.22, *P*<0.001)
Hypertension, n (%)				
No	1196 (71.1%)	142 (87.7%)		
Yes	487 (28.9%)	20 (12.3%)	0.35 (0.21-0.56, *P*<0.001)	0.36 (0.22-0.61, *P*<0.001)
Diabetes				
No	1542 (91.6%)	147 (90.7%)		
Yes	141 (8.4%)	15 (9.3%)	1.12 (0.64-1.95, *P*=0.700)	
Hepatitis_B				
No	1629 (96.8%)	155 (95.7%)		
Yes	54 (3.2%)	7 (4.3%)	1.36 (0.61-3.05, *P*=0.451)	
Tumor_history				
No	1644 (97.7%)	157 (96.9%)		
Yes	39 (2.3%)	5 (3.1%)	1.34 (0.52-3.45, *P*=0.541)	
CKpan				
Negative	3 (0.2%)	2 (1.2%)		
Partially positive	59 (3.5%)	9 (5.6%)	0.23 (0.03-1.56, *P*=0.133)	0.11 (0.00-6.16, *P*=0.283)
Positive	1621 (96.3%)	151 (93.2%)	0.14 (0.02-0.84, *P*=0.032)	0.08 (0.00-4.15, *P*=0.210)
P40				
Negative	45 (2.7%)	6 (3.7%)		
Partially positive	250 (14.9%)	24 (14.8%)	0.72 (0.28-1.86, *P*=0.498)	
Positive	1388 (82.5%)	132 (81.5%)	0.71 (0.30-1.70, *P*=0.447)	
P16				
Negative	1453 (86.3%)	139 (85.8%)		
Partially positive	191 (11.3%)	20 (12.3%)	1.09 (0.67-1.79, *P*=0.719)	
Positive	39 (2.3%)	3 (1.9%)	0.80 (0.25-2.64, *P*=0.719)	
P63				
Negative	39 (2.3%)	7 (4.3%)		
Partially positive	132 (7.8%)	11 (6.8%)	0.46 (0.17-1.28, *P*=0.138)	
Positive	1512 (89.8%)	144 (88.9%)	0.53 (0.23-1.21, *P*=0.131)	
EGFR				
Negative	6 (0.4%)	1 (0.6%)		
Partially positive	37 (2.2%)	4 (2.5%)	0.65 (0.06-6.84, *P*=0.719)	
Positive	1640 (97.4%)	157 (96.9%)	0.57 (0.07-4.80, *P*=0.609)	
EBER, n (%)				
Negative	18 (1.1%)	1 (0.6%)		
Partially positive	118 (7%)	9 (5.6%)	1.37 (0.16-11.49, *P*=0.770)	
Positive	1547 (91.9%)	152 (93.8%)	1.77 (0.23-13.34, *P*=0.580)	
EBV, n (%)				
Negative	482 (28.6%)	11 (6.8%)		
Positive	1201 (71.4%)	151 (93.2%)	5.51 (2.96-10.25, *P*<0.001)	3.66 (1.92-7.00, *P*<0.001)
Age (Mean ± SD)	52.5 ± 12.8	51.2 ± 11.7	0.99 (0.98-1.00, *P*=0.216)	
BMI (Mean ± SD)	23.8 ± 3.6	23.7 ± 3.4	0.99 (0.95-1.04, *P*=0.826)	
ALT (Mean ± SD)	24.8 ± 22.0	26.7 ± 25.4	1.00 (1.00-1.01, *P*=0.291)	
AST (Mean ± SD)	21.0 ± 13.9	22.4 ± 11.1	1.01 (1.00-1.01, *P*=0.244)	
ALB (Mean ± SD)	46.6 ± 3.7	46.0 ± 4.4	0.97 (0.93-1.01, *P*=0.102)	
GLOB (Mean ± SD)	29.3 ± 5.1	29.9 ± 6.2	1.02 (0.99-1.06, *P*=0.117)	
BUN (Mean ± SD)	5.1 ± 1.4	5.1 ± 1.5	1.02 (0.91-1.14, *P*=0.766)	
CREA (Mean ± SD)	74.3 ± 15.7	74.6 ± 15.9	1.00 (0.99-1.01, *P*=0.827)	
LDH (Mean ± SD)	165.9 ± 44.4	199.0 ± 74.1	1.01 (1.01-1.01, *P*<0.001)	1.01 (1.00-1.01, *P*<0.001)
WBC (Mean ± SD)	7.0 ± 1.9	7.2 ± 2.9	1.06 (0.99-1.15, *P*=0.101)	
HGB (Mean ± SD)	141.0 ± 14.4	139.8 ± 14.2	0.99 (0.98-1.01, *P*=0.305)	
PLT (Mean ± SD)	238.1 ± 67.1	239.6 ± 76.6	1.00 (1.00-1.00, *P*=0.786)	
NE (Mean ± SD)	4.5 ± 1.6	4.9 ± 2.7	1.12 (1.03-1.21, *P*=0.007)	1.08 (0.99-1.19, *P*=0.096)
LY (Mean ± SD)	1.8 ± 0.7	1.6 ± 0.6	0.68 (0.51-0.90, *P*=0.008)	0.67 (0.49-0.93, *P*=0.015)

**Figure 2:**
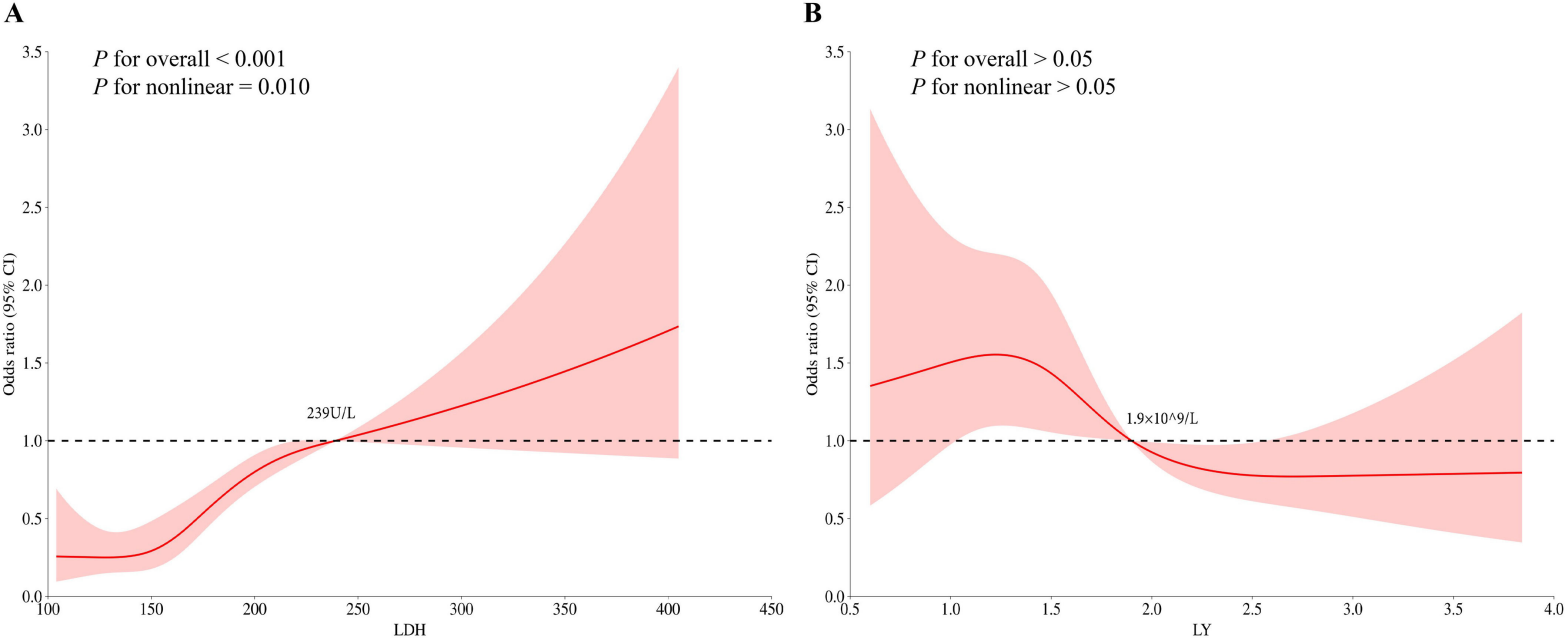
Restricted cubic spline (RCS) plots. **(A)** LDH; **(B)** LY.

Based on the findings from the multivariate logistic regression analysis, we proceeded to investigate the relationship between LDH, LY levels and the risk of distant metastasis in NPC by RCS analysis. Before examining the dose-response association, we adjusted for the potential confounding factors and conducted non-linearity assessments. The dose-response curves suggested there was a nonlinear association of LDH level with distant metastasis in NPC (*P*-overall < 0.001, *P*-non-linear = 0.010) (**Figure 2**). The risk of distant metastasis in NPC increased rapidly when the LDH level was > 239U/L. There was no significant nonlinear association of LY level with distant metastasis in NPC (*P*-overall > 0.05, *P*-non-linear > 0.05).

### 3.3 Selection of most important features and model development

**Figure 3:**
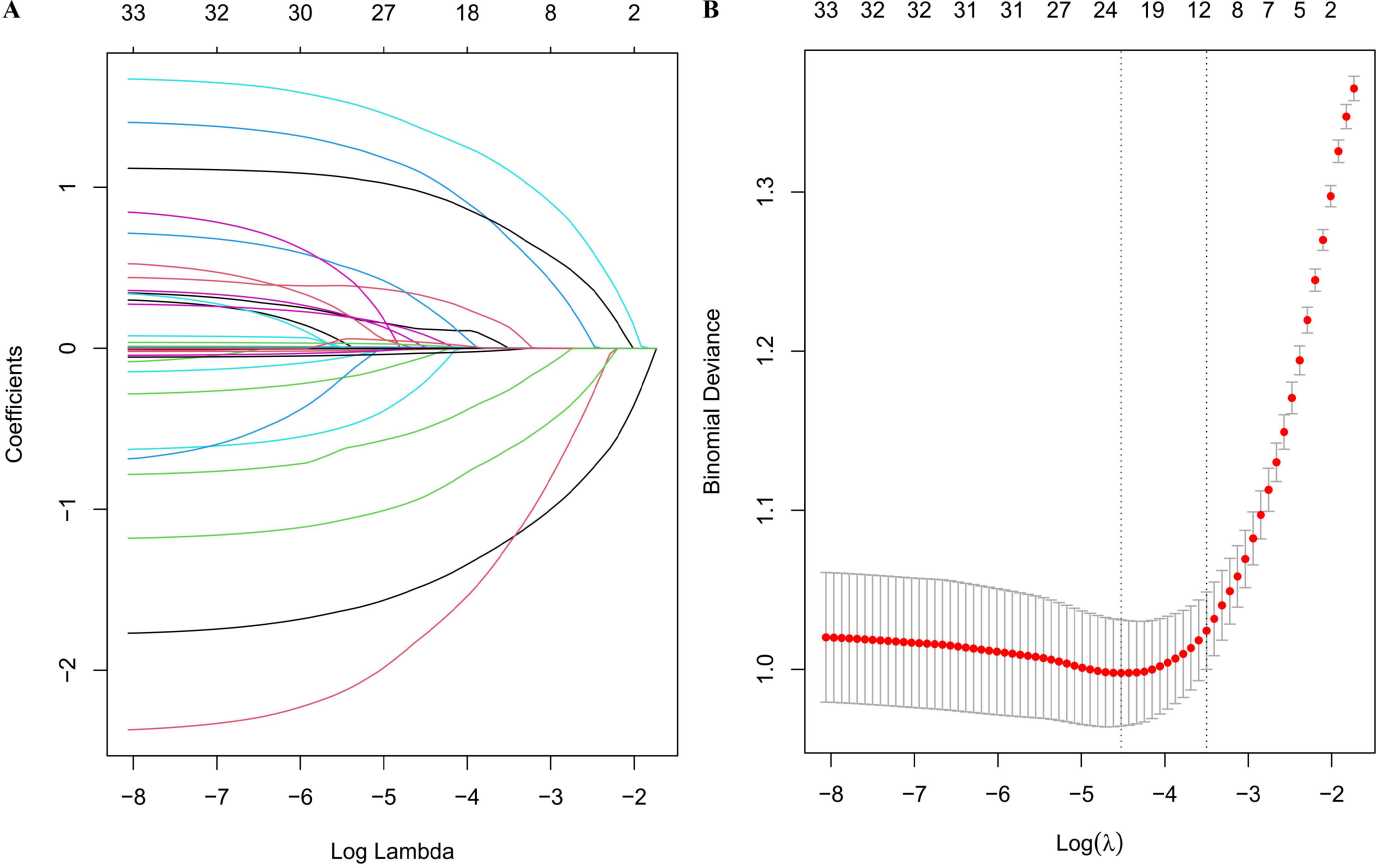
LASSO regression analysis. **(A)** LASSO regression coefficient paths; **(B)** LASSO regression cross-validation error plot.

**Figure 4:**
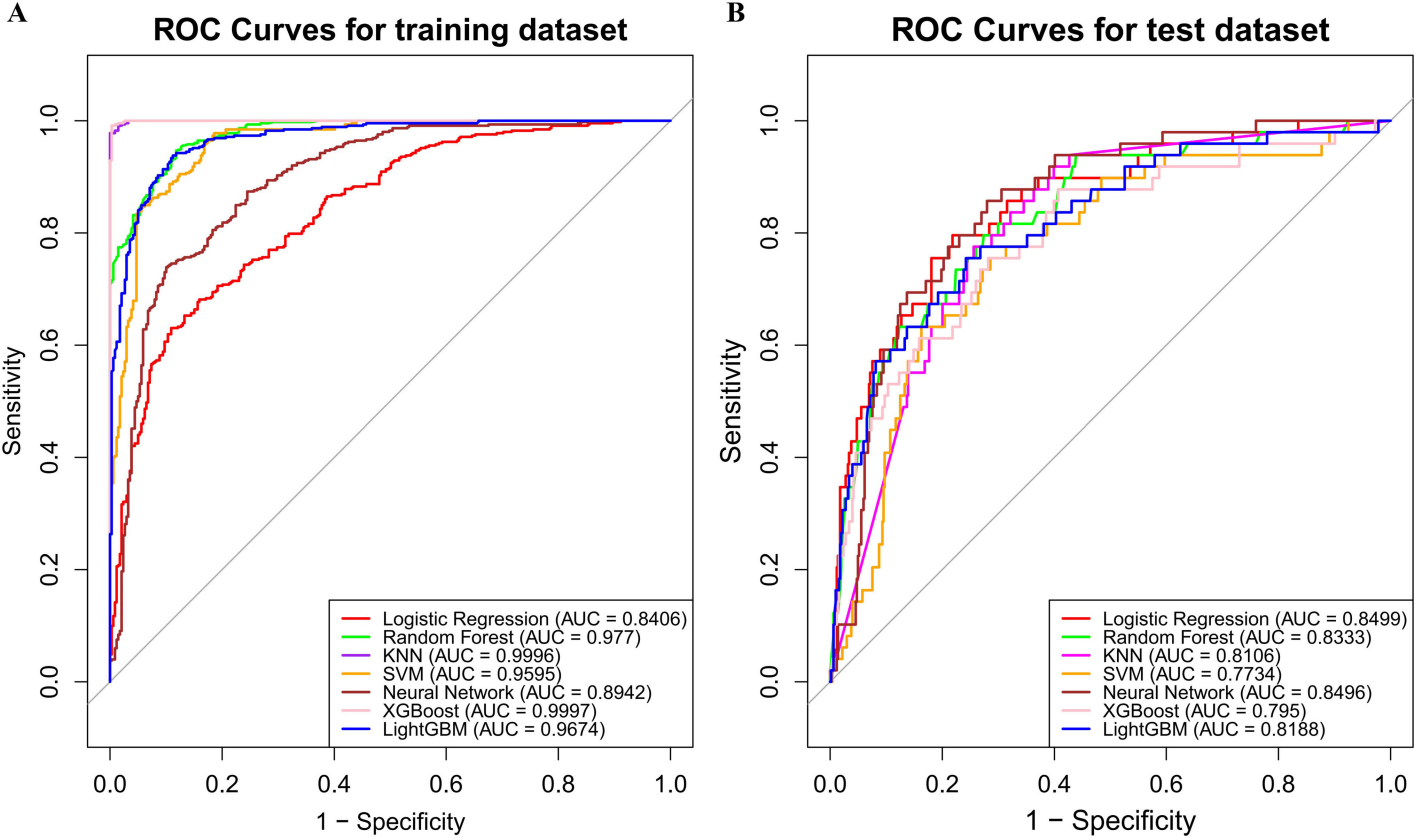
Model evaluation metrics and curves. **(A)** ROC curves for training dataset; **(B)** ROC curves for test dataset.

LASSO method was performed to screen the significant features (**Figure 3**). Eight most important features (T stage, N stage, targeted therapy, immunotherapy, hypertension, EBV, LDH and LY) were identified by LASSO regression. The ROC curves were presented in **Figure 4A** (ROC curves for training dataset) and **Figure 4B** (ROC curves for test dataset). In the test dataset, LR performed best in terms of the Area Under the ROC Curve (AUC) value (**Figure 4B**). DCA also indicated that the LR model performed best in the test dataset (**Supplementary Figure S2**). We selected the LR model to establish the predictive model for distant metastasis in NPC.

### 3.4 Model explanation

**Figure 5:**
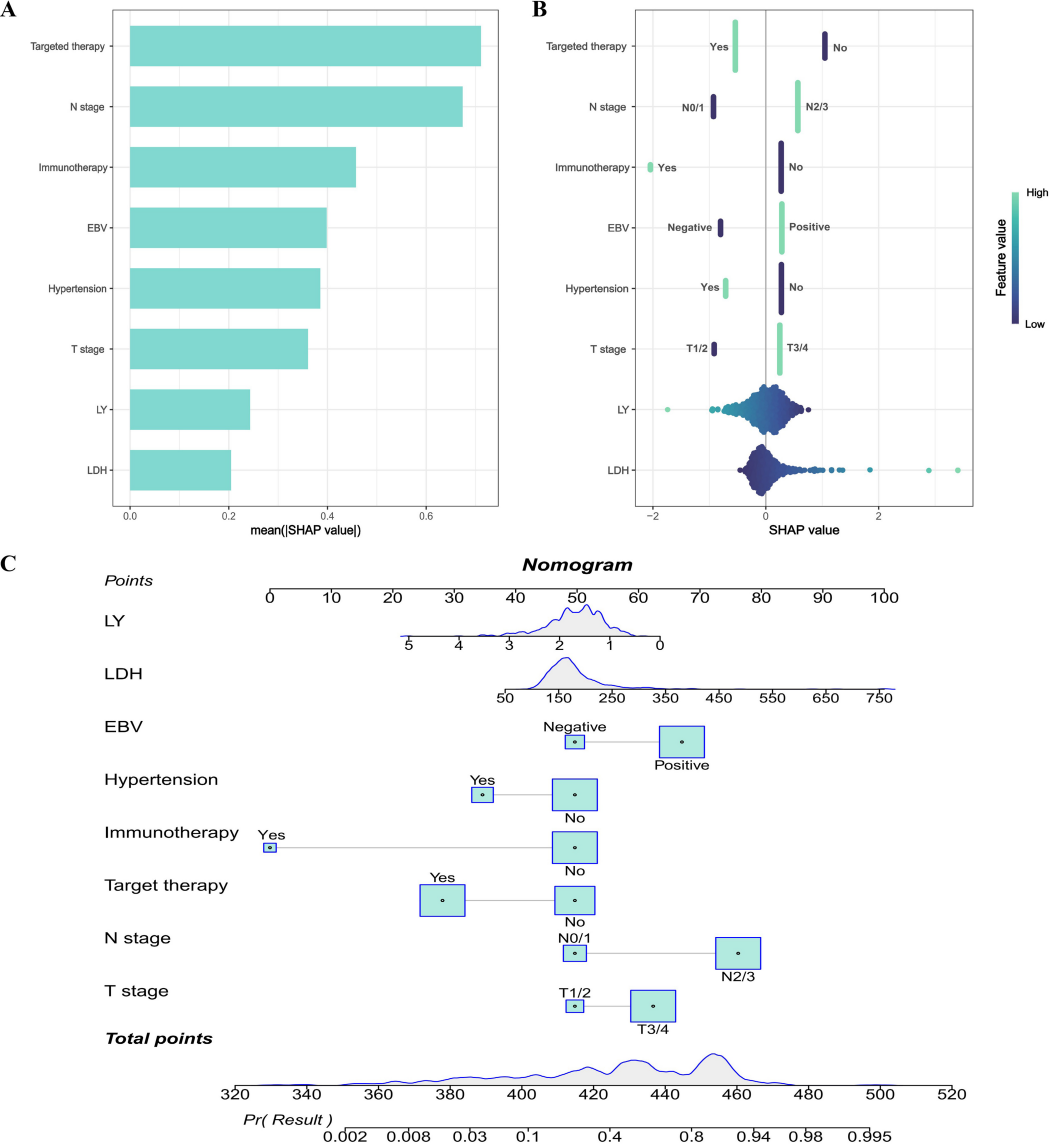
Model explanation. **(A)** SHAP summary bar plot; **(B)** SHAP summary dot plot; **(C)** Nomogram to predict the probability of distant metastasis in NPC.

We utilized the SHAP method to interpret the final model’s predictions by assessing the contribution of each feature to the forecasted outcomes. The SHAP summary bar plot illustrated the assessment of feature contributions to the model, ranked by the mean SHAP values in a descending sequence: targeted therapy, N stage, immunotherapy, EBV, hypertension, T stage, LY and LDH level (**Figure 5A**). Furthermore, the SHAP summary dot plot graphically represented the strength and direction of the impact on each feature on this model prediction (**Figure 5B**). Features including N2/3, EBV positive, T3/4 and high LDH levels were significantly associated with the increased risk of distant metastasis in NPC. On the contrary, features including targeted therapy, N0/1, immunotherapy, comorbid with hypertension, T1/2 and high level of LY could significantly reduce the risk of distant metastasis in NPC.

In addition, based on the LR model in the training dataset, a nomogram was constructed for predicting the risk of distant metastasis in NPC (**Figure 5C**). The calibration plot of LR model was shown in **Supplementary Figure S3**. To more intuitively illustrate the impact of each variable on distant metastasis in NPC, we performed a multivariate analysis on the dataset used to construct the nomogram and created a forest plot to display the results (**Supplementary Figure S4**). In the nomogram, a total score could be calculated by targeted therapy, N stage, immunotherapy, EBV, hypertension, T stage, LY and LDH level. Each of these variables was assigned a score on the point scale axis. The total score could be calculated by summing up these individual scores. By plotting the total score on the lower total point scale, we were able to estimate the likelihood of distant metastasis in NPC.

### 3.5 Sensitivity analysis

The missing data were estimated by multiple imputation for sensitivity analysis. The results of sensitivity analysis were displayed in **Supplementary Figures S5**–**S8**, which indicated that the results were robust and reliable.

## 4 Discussion

To date, although there were several studies on the prediction of the risk of distant metastasis in NPC, most of them were with small sample size (24–27). Among them, a study was based on the SEER database mining, which could not accurately represent the real circumstances of the Chinese NPC patients (27). In addition, the SEER database usually lacks some data such as comorbidities. To our knowledge, apart from the SEER database mining, this study currently represents the largest sample size examining distant metastasis in NPC. In the present study, we identified several significant risk factors of distant metastasis in NPC. Seven ML models were then used to analyze and predict the risk of distant metastasis in NPC. After comparing the performance of different ML models, the LR model performed the best and was selected to develop the prediction model for distant metastasis in NPC.

Our results identified 8 high risk factors (targeted therapy, N stage, immunotherapy, EBV, hypertension, T stage, LY and LDH level) of distant metastasis in NPC. Characteristics such as N2/3 stage, EBV positive, T3/4 stage, and elevated LDH levels were significantly correlated with an increased risk of distant metastasis in NPC. Conversely, the administration of immunotherapy and targeted therapy, along with N0/1 stage, T1/2 stage, hypertension, high level of LY were associated with a significantly reduced risk of distant metastasis in NPC. These factors could serve as important reference indicators for clinicians to assess the risk of distant metastasis in NPC patients, helping to identify high-risk populations and implement early interventions. Considering these factors in combination may have greater predictive value than considering any single factor alone and may more accurately reflect the patient’s risk of distant metastasis.

For high-risk patients, early intervention measures (such as immunotherapy and targeted therapy) might have potential benefits. Immunotherapy such as PD-1 inhibitors could suppress tumor through various mechanisms, including enhancing anti-tumor immune responses, synergistic effects of combination therapies, modulating the tumor microenvironment, and impacting tumor metastasis (28). For patients with advanced NPC, immunotherapy could improve their prognosis. The combination of PD-1 inhibitors and chemotherapy is the first-line treatment for metastatic NPC. In this study, although few patients (246 cases) received immunotherapy, only 2 of them developed distant metastasis. Therefore, for NPC patients with high risk of distant metastasis, early intervention with immunotherapy (PD-1 inhibitor) could reduce the risk of distant metastasis. In addition to immunotherapy, targeted therapy could also significantly reduce the risk of distant metastasis for NPC. Studies have shown that over 90% of NPC patients overexpressed the epidermal growth factor receptor (EGFR), and high expression of EGFR was closely associated with the aggressiveness, metastasis, resistance to radiotherapy and chemotherapy, and poor prognosis of NPC (29). Drugs such as nimotuzumab could selectively inhibit the proliferation of tumor cells by targeting EGFR, thereby improving prognosis (30). Future research could further explore the specific efficacy and safety of these early interventions in different risk groups.

Interestingly, our study indicated that NPC patients with hypertension had a lower risk of distant metastasis, with an OR value of 0.35 (0.21-0.56, *P*<0.001) in the univariate analysis, and an OR value of 0.36 (0.22-0.61, *P*<0.001) in the multivariate analysis. Some studies reported that hypertension was a risk factor for several types of cancer such as renal cell carcinoma and early cervical cancer (31, 32). A study indicated that hypertension was related to the increased risk of EBV reactivation in NPC (33). In addition, a previous study indicated that captopril could inhibit the lung tumor growth and metastasis (34). Given the high collinearity between hypertension and the use of antihypertensive medications, hypertension in this study should refer to the use of antihypertensive medications. However, which type of antihypertensive agents could reduce the risk of distant metastasis in NPC and the specific mechanisms still require further research. We will conduct a prospective study and in-depth mechanistic explorations in the future to further elucidate this issue.

There are several limitations in the present study. Firstly, this study was conducted retrospectively in a single center. This limits generalizability. Despite the strict criteria of inclusion and exclusion, there remained a challenge in eliminating potential biases that could influence the research outcomes. We are considering temporal validation to further assess the model’s performance over time. Multicenter prospective studies with large sample size are also needed in the future. Secondly, because it is a retrospective study, some important information is missing, such as drug details in complicated diseases and the time of distant metastasis. For example, for patients with hypertension, we could only know that almost all patients had taken antihypertensive drugs, but for most patients, the specific type of antihypertensive medication was not recorded. We were unable to draw Kaplan-Meier curves for metastasis-free survival stratified by risk groups based on the nomogram. Thirdly, the model is developed by using the data of Chinese patients, whether it could be used for patients in other populations still needs further study. Moreover, time is an important factor to consider, as some patients who have not experienced distant metastasis now may develop it in the future. Therefore, prospective studies are needed for more in-depth research in the future. In addition, although machine learning models possess substantial theoretical predictive capabilities, their implementation in practical clinical environments encounters various challenges such as clinicians’ understanding and trust in model outputs, and the effective utilization of models in real-time clinical decision-making processes. Furthermore, the LR model performed the best in this study (AUC = 0.8499). Although this performance metric is quite satisfactory, the AUC value might be influenced by factors such as the distribution of features in the dataset and the sample size. In future clinical practice, it is necessary to continuously update and optimize the predictive models to adapt to the evolving medical knowledge and technological advancements, thereby providing more precise support for the individualized treatment of NPC patients. Despite these limitations, the outstanding performance of our final prediction model remains undiminished.

## 5 Conclusion

In conclusion, targeted therapy, immunotherapy, N stage, EBV, hypertension, T stage, LY and LDH levels are significantly related to the risk of distant metastasis in NPC patients and could be used to identify high-risk populations for the distant metastasis in NPC. The identification of these risk factors could help clinicians develop more precise treatment plans based on individual patient characteristics, thereby improving therapeutic outcomes and reducing the risk of distant metastasis. With the implementation of multicenter studies, the identification of new features, and the application of more advanced machine learning technologies, it is anticipated that the predictive ability and intervention outcomes for distant metastasis in NPC will be further enhanced, thereby improving patient prognosis.

## Data Availability

The raw data presented in the study are included in the article/**Supplementary Material**. Any other data supporting the conclusions of this article will be made available by the authors, without undue reservation.
