# Supplementary material for: Machine learning for predicting distant metastasis in nasopharyngeal carcinoma patients
Source: Front Immunol. 2025 Jun 5;16:1580200. doi: 10.3389/fimmu.2025.1580200 (PMC12176861; doi:10.3389/fimmu.2025.1580200)

**Supplementary Figures**

**FIGURE S1** The overview of missing data for each variable. (A) Number of missing data; (B) Percentage of missing data.


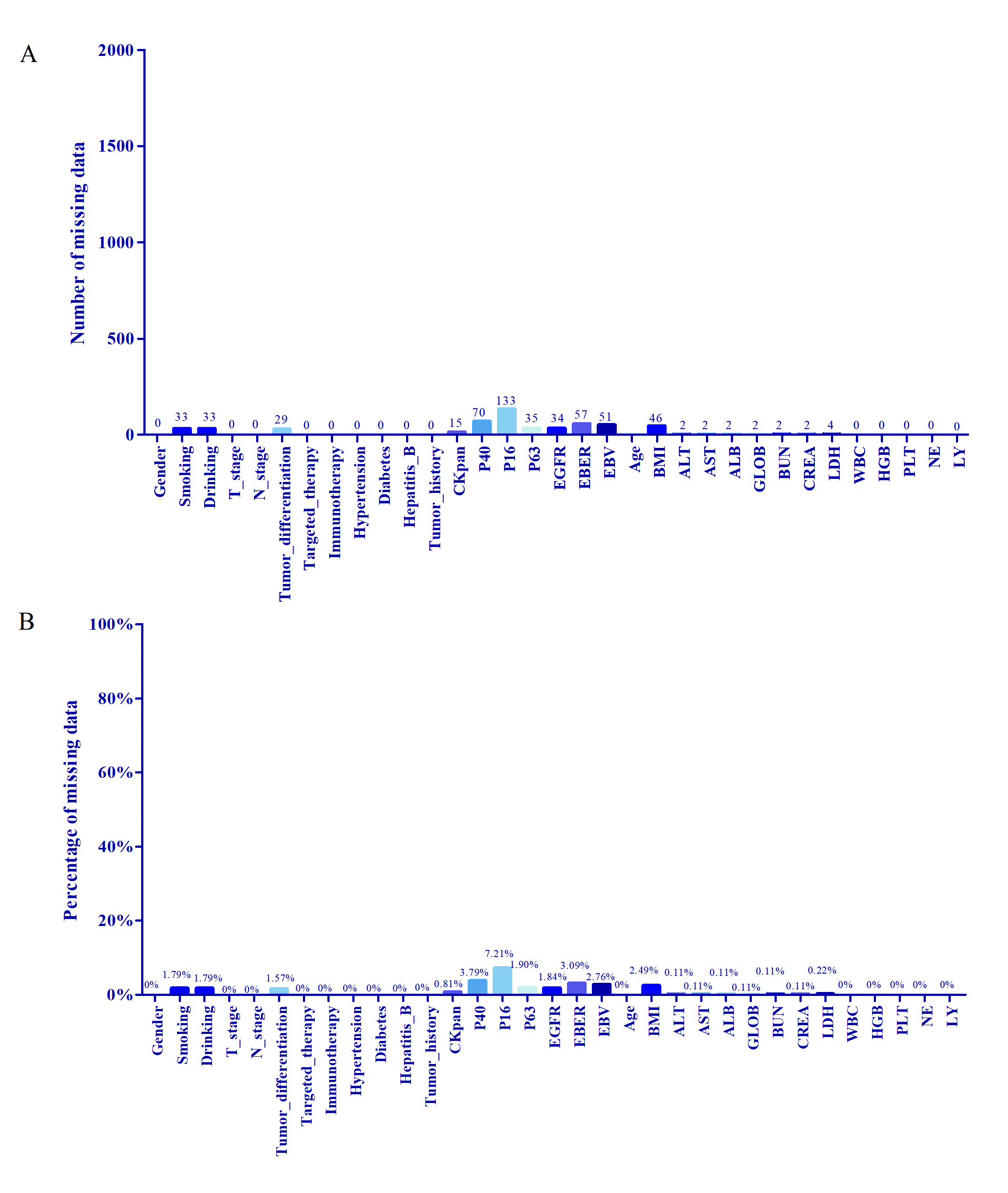


**FIGURE S2** DCA curves. (A) For training dataset; (B) For test dataset.


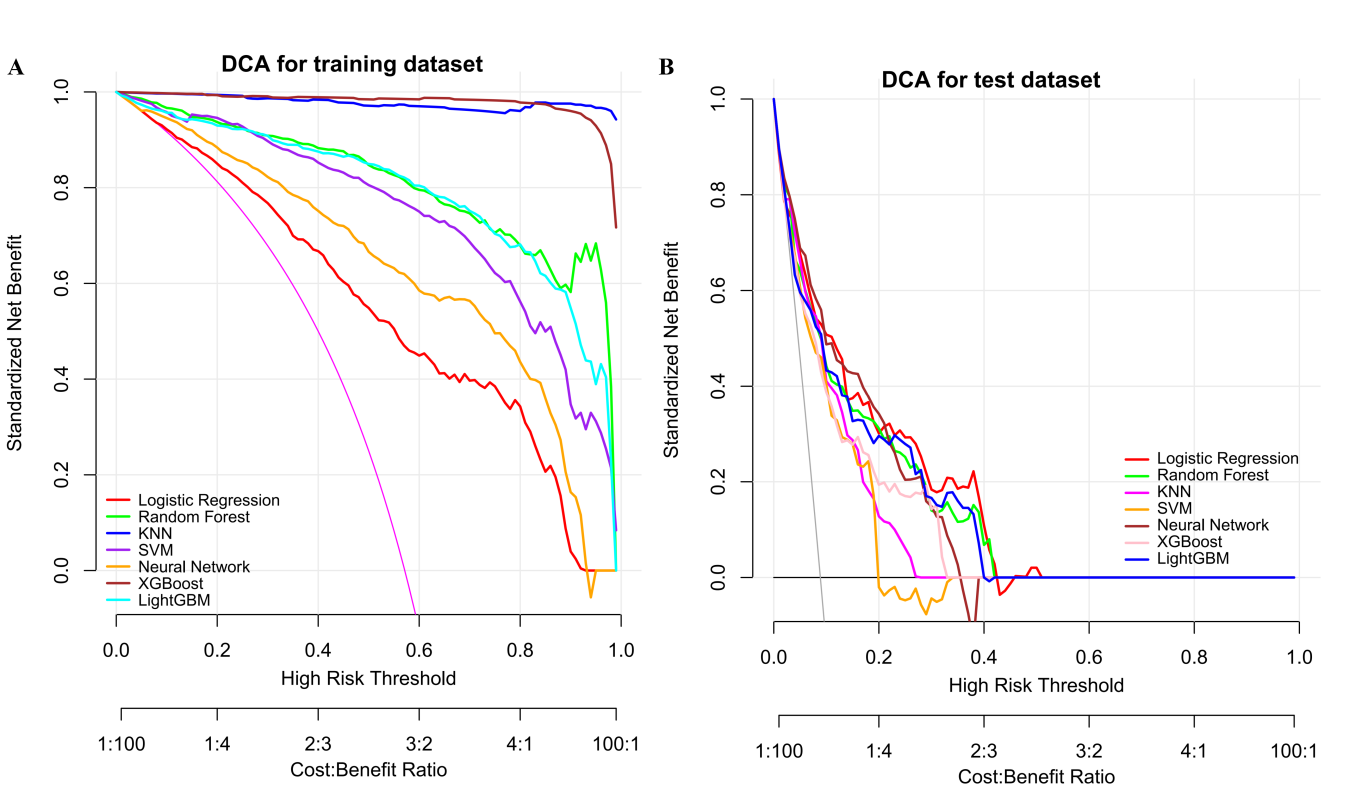


**FIGURE S3** Calibration plots and Brier scores for LR model.


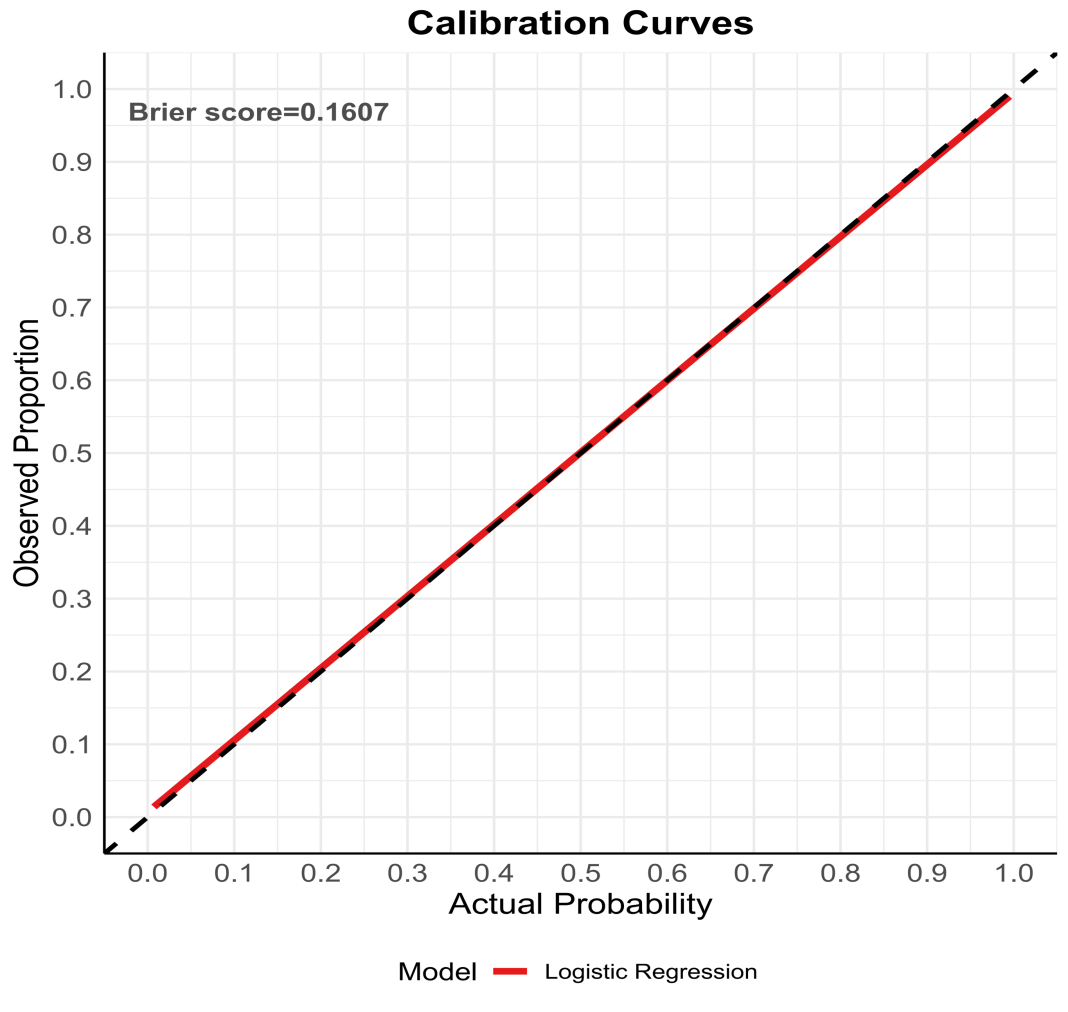


**FIGURE S4** Forest plot of each variable.


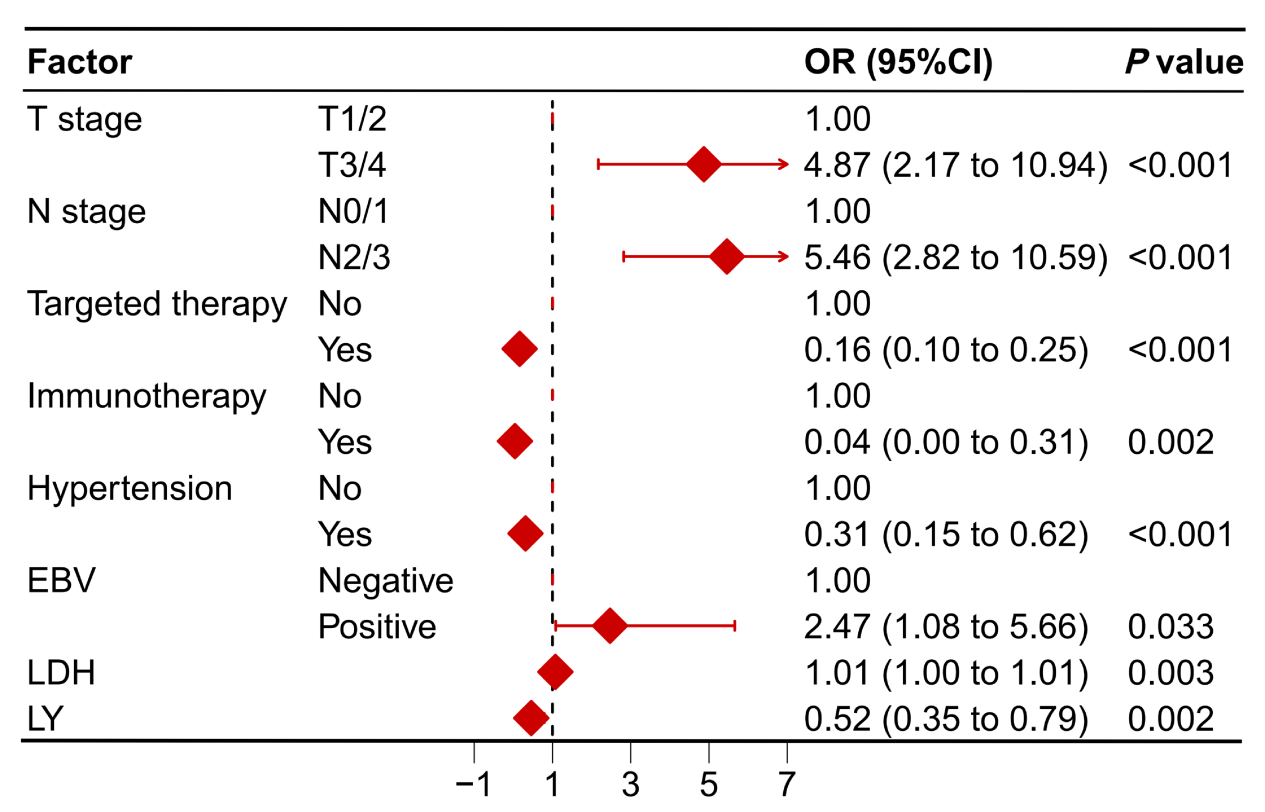


**FIGURE S5** ROC curves in the sensitivity analysis. (A) For training dataset; (B) For test dataset.


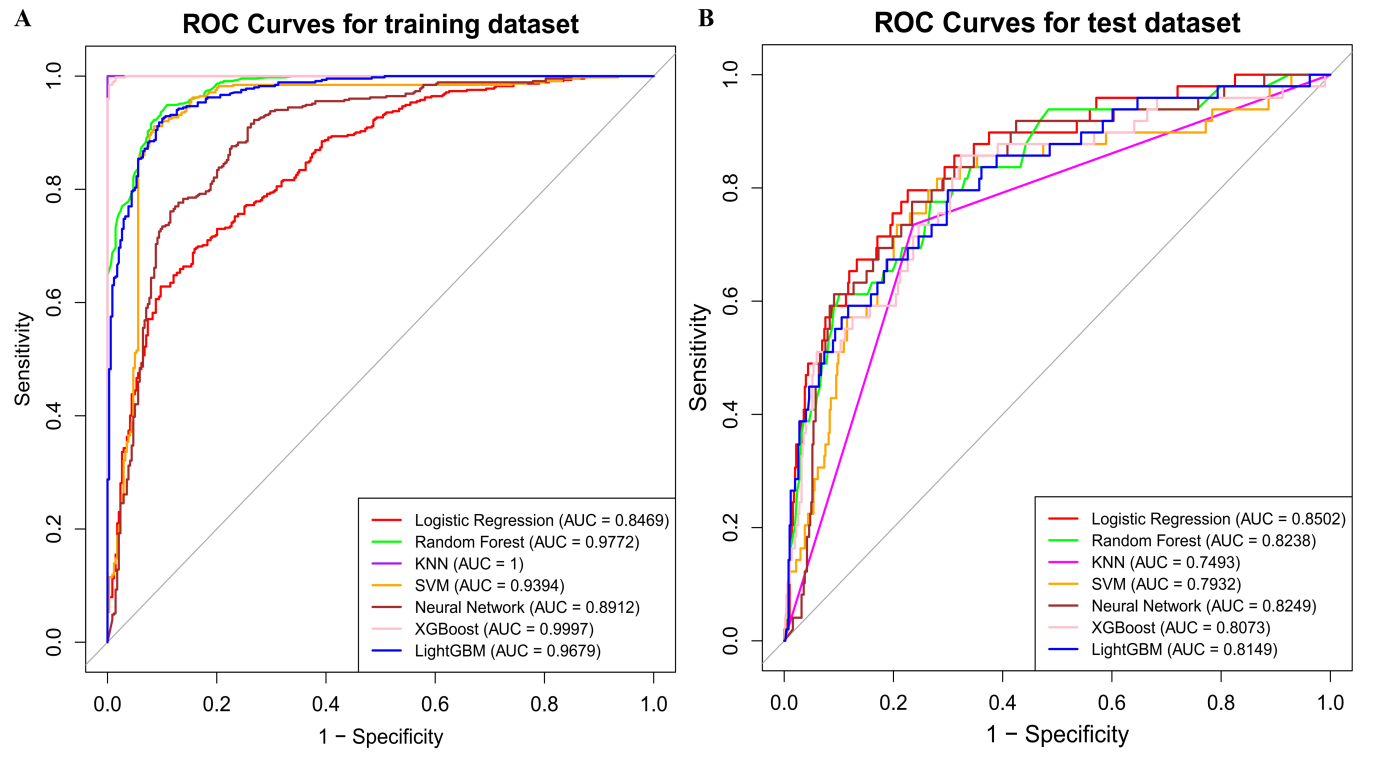


**FIGURE S6** DCA in the sensitivity analysis. (A) For training dataset; (B) For test dataset.


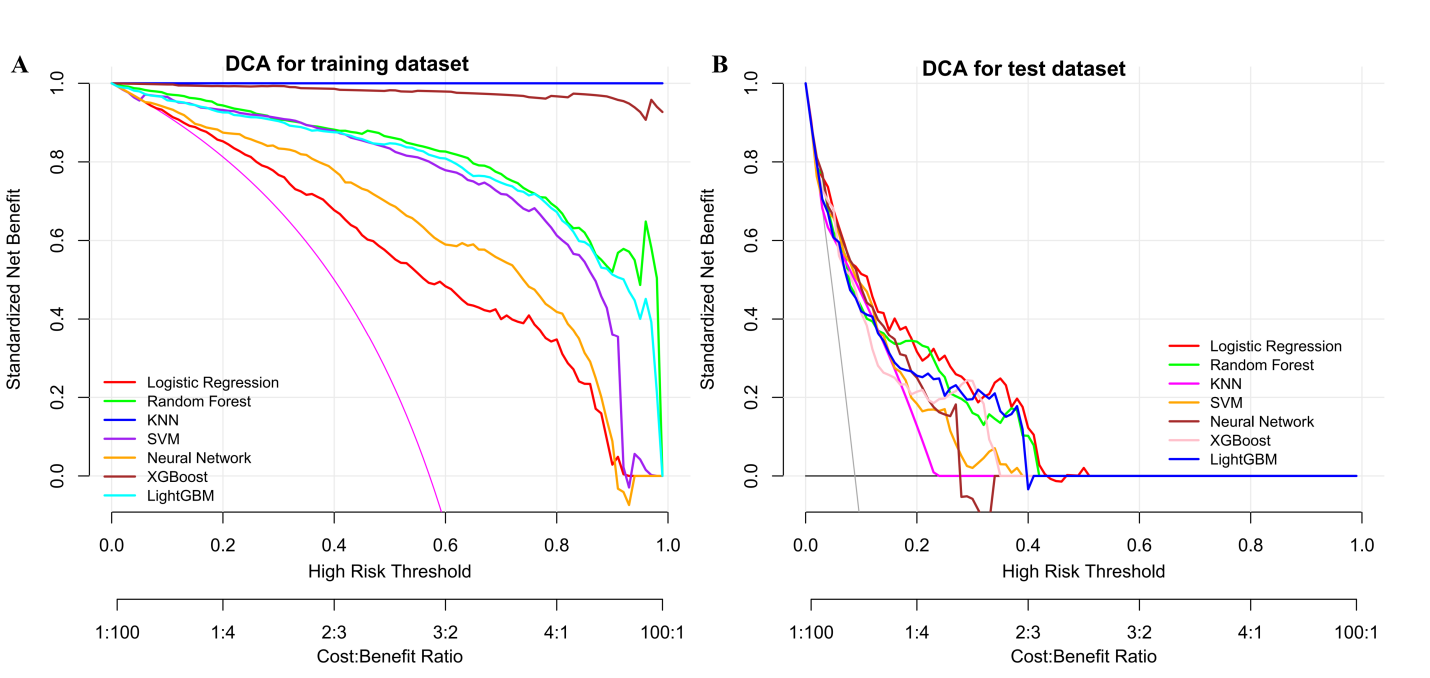


**FIGURE S7** Model explanation in the sensitivity analysis. (A) SHAP summary bar plot; (B) SHAP summary dot plot; (C) Nomogram to predict the probability of distant metastasis in NPC.


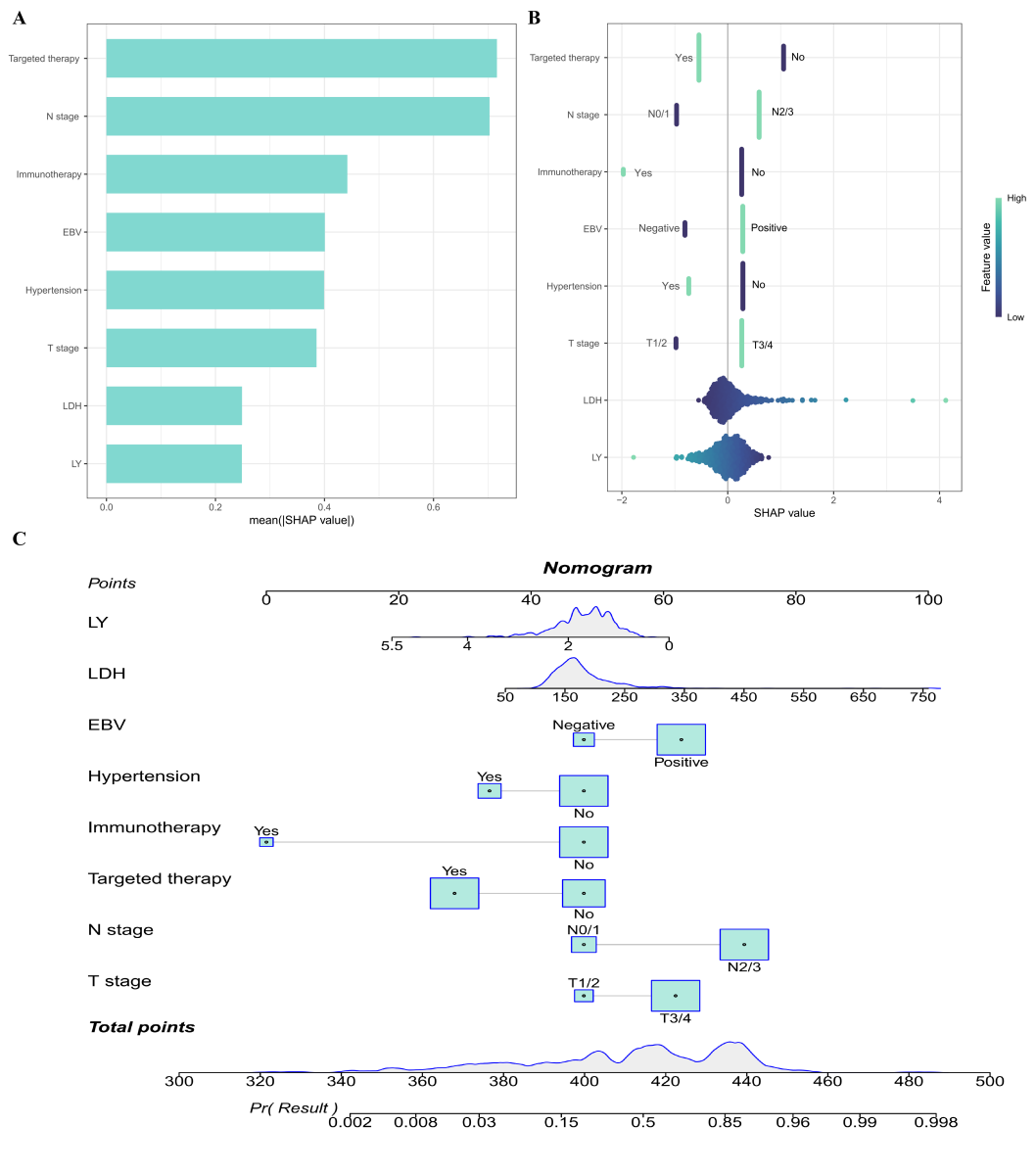


**FIGURE S8** Calibration plots and Brier scores for LR model in the sensitivity analysis.


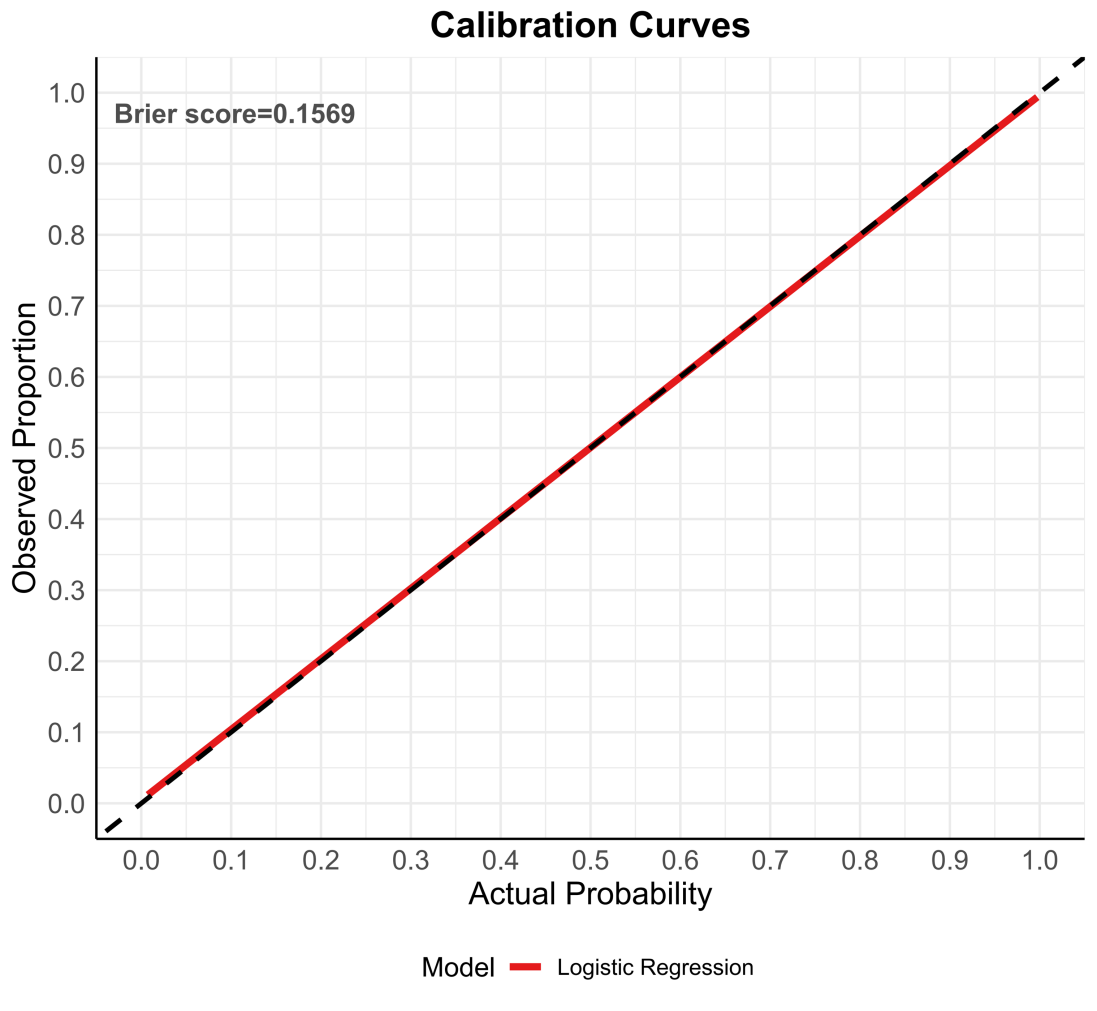

Supplement: Supplementary file 3 [file DataSheet3.docx]
