# Supplementary material for: Machine learning for predicting distant metastasis in nasopharyngeal carcinoma patients
Source: Front Immunol. 2025 Jun 5;16:1580200. doi: 10.3389/fimmu.2025.1580200 (PMC12176861; doi:10.3389/fimmu.2025.1580200)
Supplement: Supplementary file 4 [file DataSheet4.doc]

library(pbapply)

library(rlang)

library(reshape2)

library(openxlsx)

library(DALEX)

library(readr)

library(gbm)

library(dplyr)

library(caret)

library(ggplot2)

library(pROC)

library(rms)

library(rmda)

library(dcurves)

library(Hmisc)

library(ResourceSelection)

library(survey)

library(foreign)

library(plotROC)

library(shapper)

library(iml)

library(e1071)

library(ROCR)

library(corrplot)

library(lattice)

library(Formula)

library(SparseM)

library(survival)

library(riskRegression)

library(pheatmap)

library(fastshap)

library(ingredients)

library(mlr3)

library(table1)

library(tableone)

library(adabag)

library(RColorBrewer)

library(VIM)

library(mice)

library(autoReg)

library(cvms)

library(tibble)

library(data.table)

library(ComplexHeatmap)

library(circlize)

library(ROSE)

library(DMwR)

library(scales)

library(kernelshap)

library(shapviz)

library(rpart)

library(rpart.plot)

library(randomForest)

library(xgboost)

library(lightgbm)

library(kknn)

library(neuralnet)

library(NeuralNetTools)

library(gridExtra)

library(partykit)

library(missForest)

library(regplot)

library(glmnet)

library(DMwR)

library(caret)

setwd("E:/")

getwd()

log <-read.csv(file="data_NPC.csv",header = T,encoding = "GBK")

head(log)

str(log)

log$Result <- factor(log$Result,levels = c(0,1),labels = c('No','Yes'))

log$Gender <- factor(log$Gender,levels = c(0,1),labels = c('Female','Male'))

log$Smoking <- factor(log$Smoking,levels = c(0,1),labels = c('No','Yes'))

log$Drinking <- factor(log$Drinking,levels = c(0,1),labels = c('No','Yes'))

log$T_stage <- factor(log$T_stage,levels = c(0,1),labels = c('T1/2','T3/4'))

log$N_stage <- factor(log$N_stage,levels = c(0,1),labels = c('N0/1','N2/3'))

log$Tumor_differentiation <- factor(log$Tumor_differentiation,levels = c(0,1),labels = c('Undifferentiated','Differentiated'))

log$Targeted_therapy <- factor(log$Targeted_therapy,levels = c(0,1),labels = c('No','Yes'))

log$Immunotherapy <- factor(log$Immunotherapy,levels = c(0,1),labels = c('No','Yes'))

log$Hypertension <- factor(log$Hypertension,levels = c(0,1),labels = c('No','Yes'))

log$Diabetes <- factor(log$Diabetes,levels = c(0,1),labels = c('No','Yes'))

log$Hepatitis_B <- factor(log$Hepatitis_B,levels = c(0,1),labels = c('No','Yes'))

log$Tumor_history <- factor(log$Tumor_history,levels = c(0,1),labels = c('No','Yes'))

log$CKpan <- factor(log$CKpan,levels = c(0,1,2),labels = c('Negative','Partially_positive','Positive'))

log$P40 <- factor(log$P40,levels = c(0,1,2),labels = c('Negative','Partially_positive','Positive'))

log$P16 <- factor(log$P16,levels = c(0,1,2),labels = c('Negative','Partially_positive','Positive'))

log$P63 <- factor(log$P63,levels = c(0,1,2),labels = c('Negative','Partially_positive','Positive'))

log$EGFR <- factor(log$EGFR,levels = c(0,1,2),labels = c('Negative','Partially_positive','Positive'))

log$EBER <- factor(log$EBER,levels = c(0,1,2),labels = c('Negative','Partially_positive','Positive'))

log$EBV<- factor(log$EBV,levels = c(0,1),labels = c('Negative','Positive'))

missing_data <- sapply(log, function(x) sum(is.na(x)))

print(missing_data)

set.seed(123)

missForest(log)

log_imputed <- missForest(log)$ximp

print(sapply(log_imputed, function(x) sum(is.na(x))))

write.csv(log_imputed,file = "logistic_imputed_data.csv",row.names = FALSE)

log <-read.csv(file="logistic_imputed_data.csv",header = T,encoding = "GBK")

names(log)

set.seed(123)

log$Age_scaled <- scale(log$Age)[, 1]

log$BMI_scaled <- scale(log$BMI)[, 1]

log$ALT_scaled <- scale(log$ALT)[, 1]

log$AST <- scale(log$AST)[, 1]

log$ALB_scaled <- scale(log$ALB)[, 1]

log$GLOB_scaled <- scale(log$GLOB)[, 1]

log$BUN_scaled <- scale(log$BUN)[, 1]

log$CREA_scaled <- scale(log$CREA)[, 1]

log$LDH_scaled <- scale(log$LDH)[, 1]

log$WBC_scaled <- scale(log$WBC)[, 1]

log$HGB_scaled <- scale(log$HGB)[, 1]

log$PLT_scaled <- scale(log$PLT)[, 1]

log$NE_scaled <- scale(log$NE)[, 1]

log$LY_scaled <- scale(log$LY)[, 1]

head(log)

set.seed(12)

randnum <- createDataPartition(y=log$Result,

p=0.70,

list = FALSE

)

tlog<-log[randnum,]

valdata<-log[-randnum,]

tlog$Result <- as.factor(tlog$Result)

smoteData<-SMOTE(Result ~.,data=tlog, perc.over = 300, perc.under = 100)

table(smoteData$Result)

##LASSO

x <- as.matrix(smoteData[, 2:34])

y <- smoteData$Result

lasso_model <- glmnet(x, y,

family = "binomial",

alpha = 1

)

plot(lasso_model,

xvar = "lambda",

label = F

)

print(lasso_model)

set.seed(123)

cv_lasso <- cv.glmnet(x, y, family = "binomial", alpha = 1, nfolds = 10)

cv_lasso

plot(cv_lasso)

best_lambda <- cv_lasso$lambda.min

best_lambda

coef_lasso <- coef(cv_lasso, s = 0.03018 ) # s = 1se

coef_lasso

coef_lasso_matrix <- as.matrix(coef_lasso)

selected_features <- rownames(coef_lasso_matrix)[

coef_lasso_matrix[, 1] != 0

]

print(selected_features)

cat("选择的特征为：",selected_features,"\n")

coef_lasso <- coef(cv_lasso, s = 0.06) # s = 2se

coef_lasso_matrix <- as.matrix(coef_lasso)

selected_features <- rownames(coef_lasso_matrix)[coef_lasso_matrix[, 1] != 0]

print(selected_features)

selected_vars <- c("T_stage", "N_stage", "Targeted_therapy",

"Immunotherapy", "Hypertension", "EBV", "LDH", "LY")

selected_vars_scaled <- c("LDH", "LY")

smoteData$Result <- factor(smoteData$Result,levels = c(0,1),labels = c('No','Yes'))

valdata$Result <- factor(valdata$Result,levels = c(0,1),labels = c('No','Yes'))

####################ML model####################################

######LR#########

lr_model<- glm(Result ~ T_stage+N_stage+Targeted_therapy+Immunotherapy+Hypertension+EBV+LDH+LY,

data = smoteData,

family ="binomial"

)

print(lr_model)

#Brier score

#LR

smoteData$Result <- as.numeric(as.character(smoteData$Result))

predictions_lr <- predict(lr_model, newdata = smoteData, type = "response")

brier_score_lr <- mean((predictions_lr - smoteData$Result)^2)

brier_score_lr

regplot(lr_model,

title = "Nomogram",

points = TRUE,

axis.text.size = 12,

title.text.size = 14)

###################RF##########################

rf_model0 <- randomForest(Result ~ T_stage+N_stage+Targeted_therapy+Immunotherapy+Hypertension+EBV+LDH+LY,

data = smoteData,

importance=TRUE)

print(rf_model0)

set.seed(123)

ctrl <- trainControl(method = "cv",

number = 10,

search = "grid")

tuneGrid <- expand.grid(mtry = c(1:sqrt(7)))

rf_model1 <- train(Result ~ T_stage+N_stage+Targeted_therapy+Immunotherapy+Hypertension+EBV+LDH+LY,

data = smoteData,

method = "rf",

trControl = ctrl,

tuneGrid = tuneGrid

)

print(rf_model1)

rf_model1 $ bestTune

ntree_values <- seq(50, 1000, by = 50)

oob_error_rates <- numeric(length(ntree_values))

for (i in 1:length(ntree_values)) {

rf_model2 <- randomForest(Result ~ T_stage+N_stage+Targeted_therapy+Immunotherapy+Hypertension+EBV+LDH+LY,

data = smoteData,

mtry = rf_model1$bestTune$mtry,

ntree = ntree_values[i],

importance = TRUE,

oob.prox = TRUE)

oob_error_rates[i] <- rf_model2$err.rate[ntree_values[i]]

}

best_ntree <- ntree_values[which.min(oob_error_rates)]

print(paste("最佳树的数量：", best_ntree))

rf_model <- randomForest(Result ~ T_stage+N_stage+Targeted_therapy+Immunotherapy+Hypertension+EBV+LDH+LY,

data = smoteData,

ntree = best_ntree,

mtry = rf_model1$bestTune$mtry,

importance = TRUE)

print(rf_model)

########Xgboost################################

smoteData$Result <- as.numeric(smoteData$Result) - 1

valdata$Result <- as.numeric(valdata$Result) - 1

train_matrix <- xgb.DMatrix(data = as.matrix(smoteData[, selected_vars]),

label = smoteData$Result)

val_matrix <- xgb.DMatrix(data = as.matrix(valdata[, selected_vars]),

label = valdata$Result)

xgb_model0 <- xgb.train(data = train_matrix, nrounds=100)

param_grid <- expand.grid(

objective = "binary:logistic",

max_depth = c(2, 3, 4, 5),

eta = c(0.01, 0.1, 0.2),

nrounds = c(50, 100, 150)

)

best_auc <- 0

best_params <- list()

for (i in 1:nrow(param_grid)) {

param <- list(

objective = "binary:logistic",

eval_metric = "auc",

max_depth = param_grid$max_depth[i],

eta = param_grid$eta[i]

)

xgb_model_0 <- xgb.train(params = param, data = train_matrix,

nrounds = param_grid $ nrounds[i])

pred_probs <- predict(xgb_model_0, train_matrix)

roc_curve <- roc(smoteData$Result, pred_probs)

auc_value <- roc_curve$auc

if (auc_value > best_auc) {

best_auc <- auc_value

best_params <- c(param, nrounds = param_grid$nrounds[i])

}

}

print(best_params)

cat("最佳AUC: ", best_auc, "\n")

xgb_model <- xgb.train(params = best_params, data = train_matrix,

nrounds = best_params$nrounds)

print(xgb_model)

#######################LightGBM#############################

lgbtlog <- lgb.Dataset(as.matrix(smoteData[,selected_vars]),

label = smoteData$Result)

lgbvaldata <- lgb.Dataset.create.valid(lgbtlog,

as.matrix(valdata[,selected_vars]),

label = valdata$Result)

lightgbm_model0 <- lgb.train(data = lgbtlog)

param_grid <- expand.grid(

num_leaves = c(15, 31),

max_depth = c(-1, 1, 3),

learning_rate = c( 0.1, 0.2),

n_estimators = c(50),

min_data_in_leaf = c(30),

lambda_l1 = c(0, 1),

lambda_l2 = c(0, 1)

)

results <- data.frame()

for (i in 1:nrow(param_grid)) {

params <- list(

objective = "binary",

metric = "auc",

learning_rate = param_grid$learning_rate[i],

num_leaves = param_grid$num_leaves[i],

max_depth = param_grid$max_depth[i],

n_estimators = param_grid$n_estimators[i],

min_data_in_leaf = param_grid$min_data_in_leaf[i]

)

cv_results <- lgb.cv(

params = params,

data = lgbtlog,

nrounds = 10,

nfold = 5,

early_stopping_rounds = 10,

verbose = -1

)

results <- rbind(results,data.frame(param_grid[i, ],

auc=max(cv_results$record_evals$valid[['auc']]$data)))

}

best_params <- results[which.max(results$auc), ]

print(best_params)

best_params_list <- list(

objective = "binary",

metric = "auc",

learning_rate = best_params$learning_rate,

num_leaves = best_params$num_leaves,

max_depth = best_params$max_depth,

n_estimators = best_params$n_estimators,

min_data_in_leaf = best_params$min_data_in_leaf

)

lightgbm_model <- lgb.train(

params = best_params_list,

data = lgbtlog,

nrounds = best_params$n_estimators

)

print(lightgbm_model)

########################knn###########################

smoteData$Result <- factor(smoteData$Result,levels = c(0,1),labels = c('No','Yes'))

knn_model0 <- train(Result ~ T_stage+N_stage+Targeted_therapy+Immunotherapy+Hypertension+EBV+LDH_scaled+LY_scaled,

data = smoteData,

method = "kknn"

)

train_control <- trainControl(method = "cv", number = 10)

tune_grid<-expand.grid(kmax = seq(1, 20, by = 2),

distance = 2,

kernel=c("rectangular","triangular","gaussian"))

set.seed(123)

kknn_model <- train(Result ~ T_stage+N_stage+Targeted_therapy+Immunotherapy+Hypertension+EBV+LDH_scaled+LY_scaled,

data = smoteData,

method = "kknn",

trControl = train_control,

tuneGrid = tune_grid)

print(kknn_model)

best_params <- kknn_model$bestTune

print(best_params)

ggplot(kknn_model) +

theme_minimal() +

ggtitle("KNN 超参数调整结果")

knn_model <- train(Result ~ T_stage+N_stage+Targeted_therapy+Immunotherapy+Hypertension+EBV+LDH_scaled+LY_scaled,

data = smoteData,

method = "kknn",

trControl = train_control,

tuneGrid = expand.grid(kmax = best_params$kmax,

distance = best_params$distance,

kernel = best_params$kernel))

print(knn_model)

#########################SVM#######################################################

set.seed(11)

tune_result <- tune.svm(Result ~ T_stage+N_stage+Targeted_therapy+Immunotherapy+Hypertension+EBV+LDH_scaled+LY_scaled,

data = smoteData,

kernel = "radial",

cost = 10^(-1:3),

gamma = 10^(-3:1),

tunecontrol=tune.control(sampling = "cross",cross = 5),

probability = TRUE)

best_model <- tune_result$best.model

print(tune_result)

svm_model <- svm(Result ~ T_stage+N_stage+Targeted_therapy+Immunotherapy+Hypertension+EBV+LDH_scaled+LY_scaled,

data = smoteData,

kernel = "radial",

cost = best_model$cost,

gamma = best_model$gamma,

probability = TRUE)

print(svm_model)

#########################nnet#######################################################

build_nn_model <- function(hidden_layers) {

formula <- as.formula("Result ~ T_stage+N_stage+Targeted_therapy+Immunotherapy+Hypertension+EBV+LDH_scaled+LY_scaled")

model <- neuralnet(formula, data = smoteData, hidden = hidden_layers,

linear.output = FALSE)

return(model)

}

best_model_nnet <- NULL

best_auc <- 0

best_hidden_layers <- NULL

hidden_layer_combinations <- list(c(2),c(3))

for (hidden in hidden_layer_combinations) {

set.seed(123)

nn_model <- build_nn_model(hidden)

predictions_prob <- predict(nn_model, smoteData)[,2]

predictions <- ifelse(predictions_prob > 0.5, "Yes", "No")

roc_obj <- roc(smoteData$Result, predictions_prob)

auc_value <- roc_obj$auc

if (auc_value > best_auc) {

best_auc <- auc_value

best_model_nnet <- nn_model

best_hidden_layers <- hidden

}

}

cat("Best AUC:", best_auc, "\n")

cat("Best Hidden Layer Configuration:", paste(unlist(best_hidden_layers), collapse = ", "), "\n")

nnet_model <- best_model_nnet

summary(nnet_model )

#############Performance of ML models in training dataset##############################################

# LR

train_prob_lr <- predict(lr_model, newdata = smoteData,

type = 'response')

train_prob_lr

train_pred_lr <- factor(ifelse(train_prob_lr > 0.5,'Yes','No'))

train_pred_lr

# RF

train_pred_rf <- predict(rf_model, newdata = smoteData)

train_pred_rf

train_prob_rf <- predict(rf_model, newdata = smoteData,

type = "prob")[, 2]

train_prob_rf

# Xgboost

train_prob_xgb <- predict(xgb_model, train_matrix)

train_prob_xgb

train_pred_xgb <- factor(ifelse(train_prob_xgb > 0.5,'Yes','No'))

train_pred_xgb

# LightGBM

train_prob_lightgbm <- predict(lightgbm_model,

newdata = as.matrix(smoteData[, selected_vars]),

type = 'prob')

train_prob_lightgbm

train_pred_lightgbm <- predict(lightgbm_model,

newdata = as.matrix(smoteData[, selected_vars]),

type = 'class')

train_pred_lightgbm <- factor(train_pred_lightgbm,levels = c(0,1),labels = c('No','Yes'))

train_pred_lightgbm

# knn

train_pred_knn <- predict(knn_model, newdata = smoteData)

train_pred_knn

train_prob_knn <- predict(knn_model, newdata = smoteData, type = "prob")[,"Yes"]

train_prob_knn

# SVM

train_pred_svm <- predict(svm_model, newdata = smoteData)

train_pred_svm

train_prob_svm <- attr(predict(svm_model, newdata = smoteData, probability = TRUE),

"probabilities")[, "Yes"]

train_prob_svm

# NNET

train_prob_nnet <- predict(nnet_model, smoteData)[,2]

train_prob_nnet

train_pred_nnet <- factor(ifelse(train_prob_nnet > 0.5,'Yes','No'))

train_pred_nnet

#########confusion matrix####

# LR

confusion_matrix_lr <- caret::confusionMatrix(train_pred_lr,

smoteData$Result,

positive = "Yes")

print(confusion_matrix_lr)

# RF

confusion_matrix_rf <- caret::confusionMatrix(train_pred_rf,

smoteData$Result,

positive = "Yes")

print(confusion_matrix_rf)

# Xgboost

confusion_matrix_xgb <- caret::confusionMatrix(train_pred_xgb,

smoteData$Result,

positive = "Yes")

print(confusion_matrix_xgb)

# LightGBM

confusion_matrix_lightgbm <- caret::confusionMatrix(train_pred_lightgbm,

smoteData$Result,

positive = "Yes")

print(confusion_matrix_lightgbm)

# knn

confusion_matrix_knn <- caret::confusionMatrix(train_pred_knn,

smoteData$Result,

positive = "Yes")

print(confusion_matrix_knn)

# SVM

confusion_matrix_svm <- caret::confusionMatrix(train_pred_svm,

smoteData$Result,

positive = "Yes")

print(confusion_matrix_svm)

# NNET

confusion_matrix_nnet <- caret::confusionMatrix(train_pred_nnet,

smoteData$Result,

positive = "Yes")

print(confusion_matrix_nnet)

#########ROC####

# (1) lr

roc_lr <- roc(smoteData$Result,

as.numeric(train_prob_lr)

)

auc_lr <- roc_lr$auc

auc_lr

ci.auc(roc_lr)

# (2) rf

roc_rf <- roc(smoteData$Result, as.numeric(train_prob_rf))

auc_rf <- roc_rf$auc

auc_rf

ci.auc(auc_rf)

# (3) xgboost

roc_xgb <- roc(smoteData$Result, as.numeric(train_prob_xgb))

auc_xgb <- roc_xgb$auc

auc_xgb

ci.auc(auc_xgb)

# (4) lightgbm

roc_lightgbm <- roc(smoteData$Result, as.numeric(train_prob_lightgbm))

auc_lightgbm <- roc_lightgbm$auc

auc_lightgbm

ci.auc(auc_lightgbm)

# (5) knn

roc_knn <- roc(smoteData$Result, as.numeric(train_prob_knn))

auc_knn <- roc_knn$auc

auc_knn

ci.auc(auc_knn)

# (6) svm

roc_svm <- roc(smoteData$Result, as.numeric(train_prob_svm))

auc_svm <- roc_svm$auc

auc_svm

ci.auc(auc_svm)

# (7) nnet

roc_nnet <- roc(smoteData$Result, as.numeric(train_prob_nnet))

auc_nnet <- roc_nnet$auc

auc_nnet

ci.auc(auc_nnet)

# ROC

plot(roc_lr,

col = "red",

lwd = 2,

main = "ROC Curves for training dataset",

xlab = "1 - Specificity", ylab = "Sensitivity",

legacy.axes = TRUE,

cex.main = 1.5,

cex.lab = 1.2, cex.axis = 1.2

)

lines(roc_rf, col = "green", lwd = 2)

lines(roc_knn, col = "purple", lwd = 2)

lines(roc_svm, col = "orange", lwd = 2)

lines(roc_nnet, col = "brown", lwd = 2)

lines(roc_xgb, col = "pink", lwd = 2)

lines(roc_lightgbm, col = "blue", lwd = 2)

legend("bottomright",

legend = c(

paste("Logistic Regression (AUC = ", round(auc_lr, 4), ")", sep = ""),

paste("Random Forest (AUC = ", round(auc_rf, 4), ")", sep = ""),

paste("KNN (AUC = ", round(auc_knn, 4), ")", sep = ""),

paste("SVM (AUC = ", round(auc_svm, 4), ")", sep = ""),

paste("Neural Network (AUC = ", round(auc_nnet, 4), ")", sep = ""),

paste("XGBoost (AUC = ", round(auc_xgb, 4), ")", sep = ""),

paste("LightGBM (AUC = ", round(auc_lightgbm, 4), ")", sep = "") ),

col = c("red", "green", "purple", "orange",

"brown", "pink", "blue"),

lty = 1,

lwd = 2,

cex = 0.9)

############DCA######

dca_tlog <- data.frame(Result = as.numeric(smoteData$Result)-1,

train_prob_lr,

train_prob_rf,

train_prob_knn,

train_prob_svm,

train_prob_nnet,

train_prob_xgb,

train_prob_lightgbm)

# LR

dca.result_lr <- decision_curve(Result ~ train_prob_lr,

data = dca_tlog,

bootstraps = 10)

# RF

dca.result_rf <- decision_curve(Result ~ train_prob_rf,

data = dca_tlog,

bootstraps = 10)

# XGBoost

dca.result_xgb <- decision_curve(Result ~ train_prob_xgb,

data = dca_tlog,

bootstraps = 10)

# LightGBM

dca.result_lightgbm <- decision_curve(Result ~ train_prob_lightgbm,

data = dca_tlog,

bootstraps = 10)

# knn

dca.result_knn <- decision_curve(Result ~ train_prob_knn,

data = dca_tlog,

bootstraps = 10)

# SVM

dca.result_svm <- decision_curve(Result ~ train_prob_svm,

data = dca_tlog)

# NNET

dca.result_nnet <- decision_curve(Result ~ train_prob_nnet,

data = dca_tlog,

bootstraps = 10)

plot_decision_curve(

list(dca.result_lr, dca.result_rf,

dca.result_knn, dca.result_svm, dca.result_nnet,

dca.result_xgb, dca.result_lightgbm),

curve.names = c("Logistic Regression", "Random Forest",

"KNN", "SVM", "Neural Network", "XGBoost", "LightGBM"),

col = c("red", "green", "blue", "purple", "orange", "brown", "cyan", "magenta"),

lwd = 2,

confidence.intervals = FALSE,

legend.position = ("none")

)

legend("bottomleft",

legend = c("Logistic Regression", "Random Forest",

"KNN", "SVM", "Neural Network", "XGBoost", "LightGBM"),

col = c("red", "green", "blue", "purple", "orange", "brown", "cyan", "magenta"),

lwd = 2,

cex = 0.8,

bty = "n",

y.intersp = 1.3,

x.intersp = 0.8,

text.width = 0.3)

#############calibration####

calibration_data <- data.frame(

Model = rep("Logistic Regression", length(train_prob_lr)),

Probability = train_prob_lr,

Actual = as.numeric(smoteData$Result) - 1

)

ggplot(calibration_data, aes(x = Probability, y = Actual, color = Model)) +

geom_smooth(method = "lm", se = FALSE, size = 1.5) +

geom_abline(slope = 1, intercept = 0, linetype = "dashed", color = "black", size = 1) +

scale_x_continuous(limits = c(0, 1), breaks = seq(0, 1, by = 0.1)) +

scale_y_continuous(limits = c(0, 1), breaks = seq(0, 1, by = 0.1)) +

labs(

title = "Calibration Curves",

x = "Actual Probability",

y = "Observed Proportion" ) +

theme_minimal() +

theme(

plot.title = element_text(hjust = 0.5, size = 16, face = "bold"),

axis.title = element_text(size = 14),

axis.text = element_text(size = 12),

legend.position = "bottom",

legend.title = element_text(size = 12),

legend.text = element_text(size = 10),

axis.line = element_line(colour = "black")) +

scale_color_brewer(palette = "Set1")

##############################Performance of ML models in test dataset##########################

valdata$Result <- factor(valdata$Result,levels = c(0,1),labels = c('No','Yes'))

# LR

val_prob_lr <- predict(lr_model, newdata = valdata,

type = 'response')

val_prob_lr

val_pred_lr <- factor(ifelse(val_prob_lr > 0.5,'Yes','No'))

val_pred_lr

# RF

val_pred_rf <- predict(rf_model, newdata = valdata)

val_pred_rf

val_prob_rf <- predict(rf_model, newdata = valdata,

type = "prob")[, 2] #

val_prob_rf

# Xgboost

val_prob_xgb <- predict(xgb_model, val_matrix)

val_prob_xgb

val_pred_xgb <- factor(ifelse(val_prob_xgb > 0.5,'Yes','No'))

val_pred_xgb

# LightGBM

val_prob_lightgbm <- predict(lightgbm_model,

newdata = as.matrix(valdata[,selected_vars]),

type = 'prob')

val_prob_lightgbm

val_pred_lightgbm <- predict(lightgbm_model,

newdata = as.matrix(valdata[,selected_vars]),

type = 'class')

val_pred_lightgbm <- factor(val_pred_lightgbm,

levels = c(0,1),labels = c('No','Yes'))

val_pred_lightgbm

# knn

val_pred_knn <- predict(knn_model, newdata = valdata)

val_pred_knn

val_prob_knn <- predict(knn_model, newdata = valdata,

type = "prob")[,"Yes"]

val_prob_knn

# SVM

val_pred_svm <- predict(svm_model, newdata = valdata)

val_pred_svm

val_prob_svm <- attr(predict(svm_model, newdata = valdata, probability = TRUE),

"probabilities")[, "Yes"]

val_prob_svm

# NNET

val_prob_nnet <- predict(nnet_model, valdata)[,2]

val_prob_nnet

val_pred_nnet <- factor(ifelse(val_prob_nnet > 0.5,'Yes','No'))

val_pred_nnet

#########confusion matrix####

# LR

confusion_matrix_lr1 <- caret::confusionMatrix(

val_pred_lr, valdata$Result,, positive = "Yes")

print(confusion_matrix_lr1)

# RF

confusion_matrix_rf1 <- caret::confusionMatrix(val_pred_rf,

valdata$Result,,

positive = "Yes")

print(confusion_matrix_rf1)

# Xgboost

confusion_matrix_xgb1 <- caret::confusionMatrix(val_pred_xgb,

valdata$Result,,

positive = "Yes")

print(confusion_matrix_xgb1)

# LightGBM

confusion_matrix_lightgbm1 <- caret::confusionMatrix(val_pred_lightgbm,

valdata$Result,

positive = "Yes")

print(confusion_matrix_lightgbm1)

# knn

confusion_matrix_knn1 <- caret::confusionMatrix(val_pred_knn,

valdata$Result,,

positive = "Yes")

print(confusion_matrix_knn1)

# SVM

confusion_matrix_svm1 <- caret::confusionMatrix(val_pred_svm,

valdata$Result,,

positive = "Yes")

print(confusion_matrix_svm1)

# NNET

confusion_matrix_nnet1 <- caret::confusionMatrix(val_pred_nnet,

valdata$Result,

positive = "Yes")

print(confusion_matrix_nnet1)

#########ROC####

roc_lr_val <- roc(valdata$Result, as.numeric(val_prob_lr))

auc_lr_val <- roc_lr_val$auc

auc_lr_val

ci.auc(roc_lr_val)

roc_rf_val <- roc(valdata$Result, as.numeric(val_prob_rf))

auc_rf_val <- roc_rf_val $auc

auc_rf_val

ci.auc(auc_rf_val)

roc_xgb_val <- roc(valdata$Result, as.numeric(val_prob_xgb))

auc_xgb_val <- roc_xgb_val $auc

auc_xgb_val

ci.auc(auc_xgb_val)

roc_lightgbm_val <- roc(valdata$Result, as.numeric(val_prob_lightgbm))

auc_lightgbm_val <- roc_lightgbm_val $auc

auc_lightgbm_val

ci.auc(auc_lightgbm_val)

roc_knn_val <- roc(valdata$Result, as.numeric(val_prob_knn))

auc_knn_val <- roc_knn_val $auc

auc_knn_val

ci.auc(auc_knn_val)

roc_svm_val <- roc(valdata$Result, as.numeric(val_prob_svm))

auc_svm_val <- roc_svm_val$auc

auc_svm_val

ci.auc(auc_svm_val)

roc_nnet_val <- roc(valdata$Result, as.numeric(val_prob_nnet))

auc_nnet_val <- roc_nnet_val $auc

auc_nnet_val

ci.auc(auc_nnet_val)

# ROC

plot(roc_lr_val, col = "red", lwd = 2, main = "ROC Curves for test dataset",

xlab = "1 - Specificity", ylab = "Sensitivity", legacy.axes = TRUE,

cex.main = 1.6, cex.lab = 1.3, cex.axis = 1.2)

lines(roc_rf_val, col = "green", lwd = 2)

lines(roc_knn_val, col = "magenta", lwd = 2)

lines(roc_svm_val, col = "orange", lwd = 2)

lines(roc_nnet_val, col = "brown", lwd = 2)

lines(roc_xgb_val, col = "pink", lwd = 2)

lines(roc_lightgbm_val, col = "blue", lwd = 2)

legend("bottomright",

legend = c(

paste("Logistic Regression (AUC = ", round(auc_lr_val, 4), ")", sep = ""),

paste("Random Forest (AUC = ", round(auc_rf_val, 4), ")", sep = ""),

paste("KNN (AUC = ", round(auc_knn_val, 4), ")", sep = ""),

paste("SVM (AUC = ", round(auc_svm_val, 4), ")", sep = ""),

paste("Neural Network (AUC = ", round(auc_nnet_val, 4), ")", sep = ""),

paste("XGBoost (AUC = ", round(auc_xgb_val, 4), ")", sep = ""),

paste("LightGBM (AUC = ", round(auc_lightgbm_val, 4), ")", sep = "") ),

col = c("red", "green", "magenta", "orange",

"brown", "pink", "blue"),

lty = 1, lwd = 2, cex = 0.9)

############DCA######

dca_valdata <- data.frame(Result = as.numeric(valdata$Result)-1,

val_prob_lr,

val_prob_rf,

val_prob_knn,

val_prob_svm,

val_prob_nnet,

val_prob_xgb,

val_prob_lightgbm)

# Logistic Regression

dca.result_lr1 <- decision_curve(Result ~ val_prob_lr,

data = dca_valdata,

bootstraps = 10)

# Random Forest

dca.result_rf1 <- decision_curve(Result ~ val_prob_rf,

data = dca_valdata,

bootstraps = 10)

# XGBoost

dca.result_xgb1 <- decision_curve(Result ~ val_prob_xgb,

data = dca_valdata,

bootstraps = 10)

# LightGBM

dca.result_lightgbm1 <- decision_curve(Result ~ val_prob_lightgbm,

data = dca_valdata,

bootstraps = 10)

# KNN

dca.result_knn1 <- decision_curve(Result ~ val_prob_knn,

data = dca_valdata,

bootstraps = 10)

# SVM

dca.result_svm1 <- decision_curve(Result ~ val_prob_svm,

data = dca_valdata,

bootstraps = 10)

# Neural Network

dca.result_nnet1 <- decision_curve(Result ~ val_prob_nnet,

data = dca_valdata,

bootstraps = 10)

plot_decision_curve(

list(dca.result_lr1, dca.result_rf1,

dca.result_knn1, dca.result_svm1, dca.result_nnet1,

dca.result_xgb1, dca.result_lightgbm1),

curve.names = c("Logistic Regression","Random Forest",

"KNN", "SVM", "Neural Network", "XGBoost", "LightGBM"),

col = c("red", "green", "magenta", "orange","brown", "pink", "blue"),

lwd = 2, # 设置线宽

confidence.intervals = FALSE,

legend.position = ("none")

)

legend("bottomright",

legend = c("Logistic Regression","Random Forest",

"KNN", "SVM", "Neural Network", "XGBoost", "LightGBM"),

col = c("red", "green", "magenta", "orange","brown", "pink", "blue"),

lwd = 2,

cex = 0.8,

bty = "n",

y.intersp = 1.3,

x.intersp = 0.8,

text.width = 0.3)

#####################SHAP#########################################

######LR####

n_smoteData = 450

n_valdata = 450

explain_kernel_lr <- kernelshap(lr_model,

smoteData[1:n_smoteData,selected_vars],

bg_X = valdata[1:n_valdata,selected_vars])

shap_value_lr <- shapviz(explain_kernel_lr,

X_pred = smoteData[1:n_smoteData,selected_vars],

interactions = TRUE)

sv_importance(shap_value_lr,

kind = "bar",

show_numbers = F,

fill = "#81D8D0" )+

theme_bw()+

ggtitle("Logistic")+

theme(plot.title = element_text(hjust = 0.5,face = "bold",color = "black"))

sv_importance(shap_value_lr,

kind = "beeswarm",

viridis_args = list(begin = 0.25, end = 0.85, option = "G"),

show_numbers = FALSE)+

ggtitle("Logistic")+

theme_bw()+

theme(plot.title = element_text(hjust = 0.5,face = "bold",color = "black"))
